# Identification of Cumin (*Cuminum cyminum*) MicroRNAs through Deep Sequencing and Their Impact on Plant Secondary Metabolism

**DOI:** 10.3390/plants12091756

**Published:** 2023-04-25

**Authors:** Almendra Reyes-Calderón, Claudia Gutiérrez-García, Andrea G. Urióstegui-Pena, Aashish Srivastava, Liliana Aguilar-Marcelino, Hafiz M. N. Iqbal, Shiek S. S. J. Ahmed, Sujay Paul, Ashutosh Sharma

**Affiliations:** 1 Tecnologico de Monterrey, Centre of Bioengineering, NatProLab, Plant Innovation Lab, School of Engineering and Sciences, Queretaro 76130, Mexico; 2 Department of Clinical Science, University of Bergen, 5021 Bergen, Norway; 3 Centro Nacional de Investigación Disciplinaria en Salud Animal e Inocuidad, INIFAP, Jiutepec 62550, Mexico; 4 Tecnologico de Monterrey, School of Engineering and Sciences, Campus Monterrey, Ave. Eugenio Garza Sada 2501, Monterrey 64849, Mexico; 5 Drug Discovery and Multi-Omics Laboratory, Faculty of Allied Health Sciences, Chettinad Hospital and Research Institute, Chettinad Academy of Research and Education, Kelambakkam 603103, India

**Keywords:** microRNA (miRNA), high-throughput sequencing, gene regulation, pharmacogenomics, medicinal plant, secondary metabolites, *Cuminum cyminum*

## Abstract

The pharmacological properties of plants lie in the content of secondary metabolites that are classified into different categories based on their biosynthesis, structures, and functions. MicroRNAs (miRNAs) are small non-coding RNA molecules that play crucial post-transcriptional regulatory roles in plants, including development and stress-response signaling; however, information about their involvement in secondary metabolism is still limited. Cumin is one of the most popular seeds from the plant *Cuminum cyminum*, with extensive applications in herbal medicine and cooking; nevertheless, no previous studies focus on the miRNA profile of cumin. In this study, the miRNA profile of *C. cyminum* and its association with the biosynthesis of secondary metabolites were determined using NGS technology. The sequencing data yielded 10,956,054 distinct reads with lengths ranging from 16 to 40 nt, of which 349 miRNAs were found to be conserved and 39 to be novel miRNAs. Moreover, this work identified 1959 potential target genes for *C. cyminum* miRNAs. It is interesting to note that several conserved and novel miRNAs have been found to specifically target important terpenoid backbone, flavonoid biosynthesis, and lipid/fatty acid pathways enzymes. We believe this investigation will aid in elucidating the implications of miRNAs in plant secondary metabolism.

## 1. Introduction

*Cuminum cyminum* L. (Apiaceae), commonly known as cumin, is an annual, aromatic, herbaceous plant [1,2,3,4] native to Egypt, the Mediterranean, and the countries of South Asia. This plant has been widely grown in India, China, Turkey, Pakistan, Morocco, Egypt, Syria, Mexico, Iran, and Chile [5]. Cumin is a commercially important seed spice known for its distinct aromatic impact in a variety of culinary cultures around the world [4,5,6,7,8], pharmacological attributes in both traditional and current medicine [1,3,5,6], as well as in beverage, perfumery, alimentary [4,5], and beauty industries [2]. Asia, the Middle East, and North Africa have been identified as the largest producers and exporters of cumin seeds and products [4]. The world production of cumin in 2012 was approximately 300,000 tons, with approximately 70% of the production coming from India. The main import markets for cumin seeds are the United States (15.8%), Egypt (13%), Brazil (6%), the United Kingdom (5.6%), and Spain (5.5%) [5]. Dietary cumin provides high amounts of fat, protein, dietary fiber, vitamins B, C, E, and minerals, especially iron [5,6]. Moreover, *C. cyminum* contains a variety of bioactive compounds, the most prominent of which are alkaloids, flavonoids, and terpenoids [1,4]. Cuminaldehyde, cymene, cumin alcohol, and other terpenoids are the main volatile biomolecules found in *C. cyminum* essential oil [6,8]. Furthermore, cumin seeds are traditionally used to treat stomach aches, dyspepsia, indigestion, flatulence, toothache, hypertension, scorpion stings, obesity, jaundice, vomiting, chronic diarrhea, fever, ulcers, and cold in different countries [1,3,5,6].

MicroRNAs (miRNAs) are short non-coding RNA sequences (20–24 nucleotides) [9,10,11,12] found in animals, plants, and some viruses [12,13,14] that regulate gene expression at the post-transcriptional level [12,14] through mRNA inhibition. In plants, miRNAs are synthesized by RNA polymerase II (Pol II) as a primary transcript (pri-miRNA) and then processed by a DICER-LIKE endonuclease complex 1 (DCL1) to form the mature miRNA [9,10,11,13,14]. Interestingly, these tiny molecules are found in all major plant lineages and can be grouped into two classes: (a) highly expressed and evolutionarily conserved miRNAs and (b) non-conserved/species specific/novel miRNAs that expressed at low levels or induced under specific conditions [9]. Plant miRNAs largely control transcription factors and play an important role in plant growth and development, as well as abiotic and biotic stress response signaling [9,11,14,15]. Intriguingly, some of the previous research has also shown that a number of microRNAs are involved in secondary plant metabolism [9,14]. For instance, Barozai and colleagues (2013) identified 17 novel miRNAs in carrots, out of which five were involved in plant metabolism [16], while microRNAs miR5140, miR159, miR172, miR5303, miR5658, miR477, miR1426, miR530, and miR5079 from *Withania somnifera* (Indian ginseng) were found to be associated with the biosynthesis of steroid, stilbenoid, diarylheptanoid, gingerol, phenylpropanoid, isoquinoline, glycosyltransferases, ubiquinone, carotenoid, and other diterpenoids [17]. Similarly, miR4995, miR5021, and miR6300 from the medicinal plant *Cinnamomum camphora* are linked to the regulation of the biosynthesis of highly valuable terpenoids [18]. MicroRNAs from tree peony (*Paeonia* sp.) seeds such as miR414, miR156b, miR2673b, miR7826, and novel-m0027-5p were also reported to be associated with the fatty acids and lipid metabolism through post-transcriptional regulation [19]. In using high-throughput sequencing technology, six miRNAs (miRNAs miR164d, miR168b, novelmiRNA-108, novelmiRNA-23, novelmiRNA-58, and novelmiRNA-191) potentially involved in lipid biosynthesis were identified in medicinal plant Sea buckthorn (*Hippophae* L.). Recently, Gutiérrez-García et al. (2022) identified several miRNAs in the leaves of the medicinal plant Curry (*Murraya koenigii*), such as mko-miR5082, mko-miR396g-5p, mko-miRN7-3p, mko-miR827b, mko-mir156, mko-mir396c, mko-miRN4-5p, mko-miR168b, mko-miR858, mko-miR8610.1, and mko-miR5082. These are involved in the biosynthesis of terpenoids and flavonoids [20].

It has been suggested that artificial miRNAs (amiRNAs) can be used to manipulate target transcripts in order to increase secondary metabolite production *in planta*. However, a deeper comprehension of their mechanisms of action is necessary for utilizing them in the metabolic engineering process. Therefore, the current study aims to generate a secondary metabolism biosynthesis pathway-associated miRNA profile from *C. cyminum* using NGS technology.

## 2. Results

### 2.1. Sequence Analysis of C. cyminum Small RNAs

**Figure 1:**
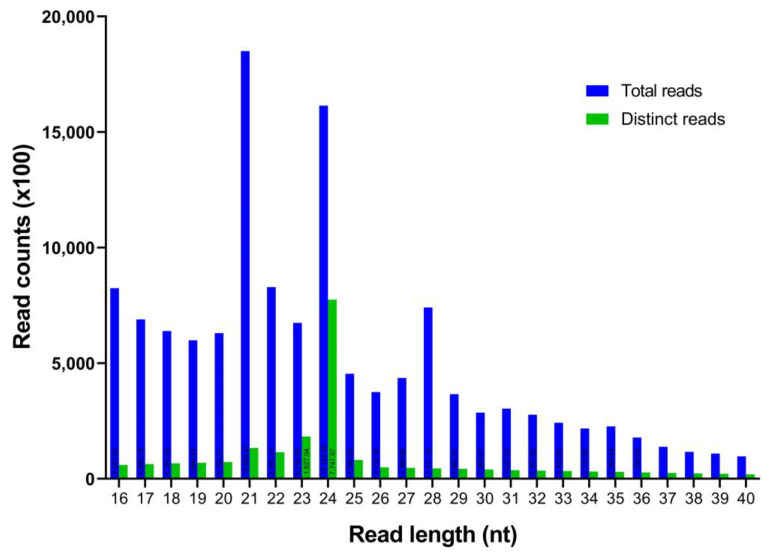
Length distribution and abundance of the cumin small RNA sequences obtained by Illumina sequencing.

In this work, a total of 17,058,681 raw reads from the *C. cyminum* seedling samples were obtained (NCBI SRA accession number: SRP376517). Subsequently, the adaptors, low-quality reads, and other short RNAs like rRNA, snoRNA, snRNA, and tRNA were removed (Table 1), acquiring a total of 2,126,409 distinct reads with lengths ranging from 16 to 40 nt. According to the size distribution of unique *C. cyminum* reads, 24 nt was found to be the most prevalent one (36.44%), followed by 23 nt (8.59%), 21 nt (6.25%), and 22 nt (5.40%) (Figure 1).

### 2.2. Identification of Conserved and Novel miRNAs in C. cyminum

**Figure 2:**
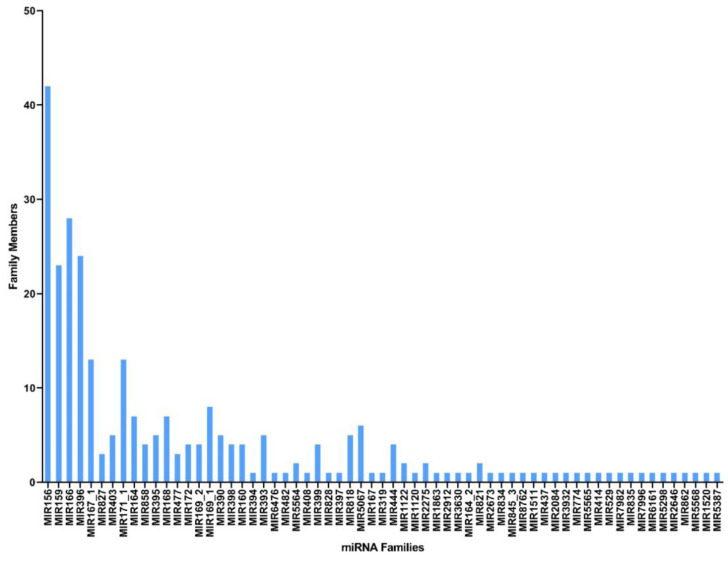
miRNA families with the corresponding number of members found in *C. cyminum*.

Unique reads obtained in this study were first matched against miRbase-22 (https://www.mirbase.org/ accessed on 10 January 2022) using the BLASTn program, and a total of 142 conserved cumin miRNAs spanning 63 miRNA families were identified (Table 2 shows the conserved miRNAs with the highest read counts for each family; for further info, please refer to Supplementary Materials). The frequency of the conserved miRNAs varied greatly between families, and all showed substantial homology with their respective homologs (maximum of single mismatch). Conserved miRNA data showed that the most abundant miRNA families were miR156, miR166, miR396, and miR159, with 42, 28, 24, and 23 members, respectively (Figure 2); moreover, the read counts of the miRNA families varied from 1 to 400,512, where the miR156 family had the highest number of reads (400,512), followed by the miR159 (184,400) and miR166 (144,536) families (Supplementary Table S1).

**Figure 3:**
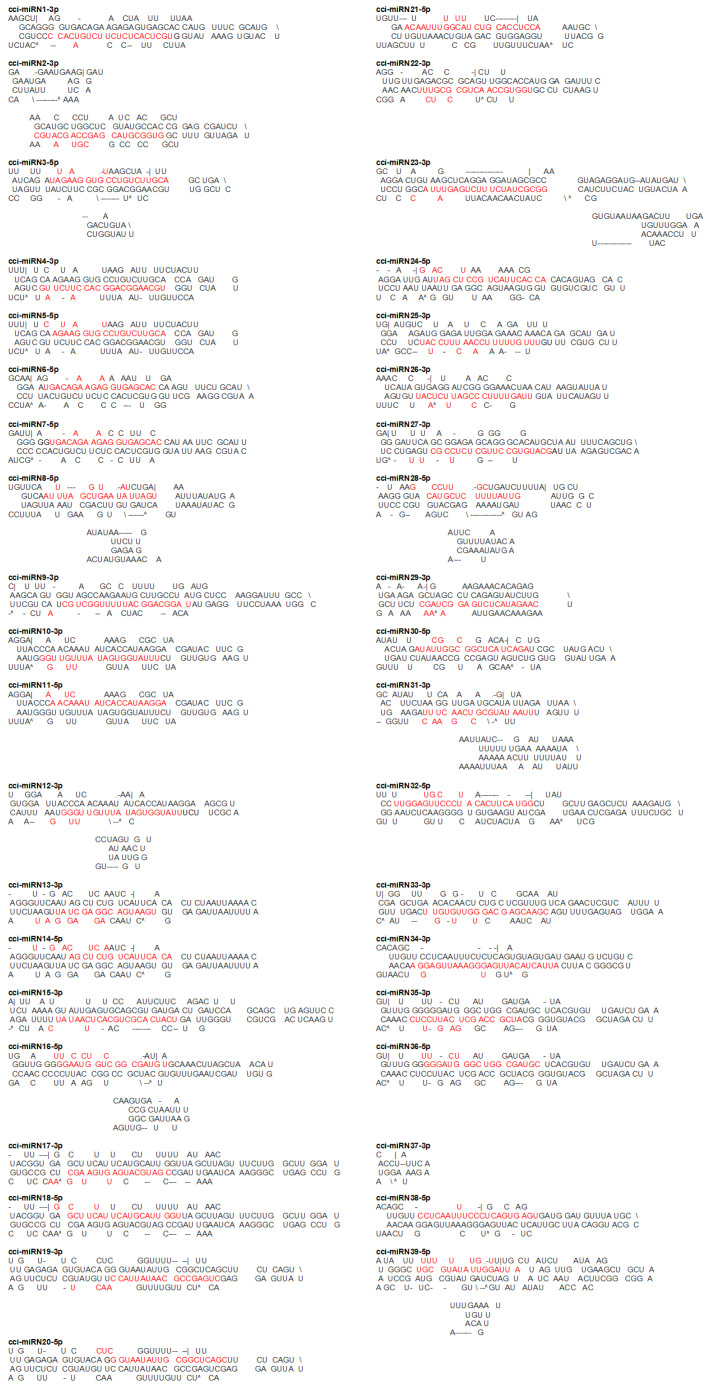
Secondary structures (stem-loops) of *C. cyminum* novel miRNA precursors. Mature miRNAs are highlighted in red font.

Following the identification of conserved miRNAs, the remaining unaligned 1,057,102 reads were subjected to predict novel *C. cyminum* miRNA candidates by applying strict filtering criteria. A total of 39 novel cumin miRNAs were identified in this study (Table 3), and their secondary hairpin structures were deduced (Figure 3). The read counts of these novel miRNA candidates varied from 2 to 1382. The cci-miRN10-3p displayed the highest number of reads (1382), followed by cci-miRN2-3p (975) and cci-miRN20-5p (262). The precursor sequences of these novel miRNA candidates had high Minimum Folding Free Energy Index (MFEI) values ranging from 0.70 to 1.41 with an average of 0.98 ± 0.14, distinguishing them from other types of RNAs such as tRNAs (0.64), rRNAs (0.59), and mRNAs (0.62–0.66) [21].

### 2.3. Target Prediction of Conserved and Novel C. cyminum miRNAs and Their Functional Analysis

**Figure 4:**
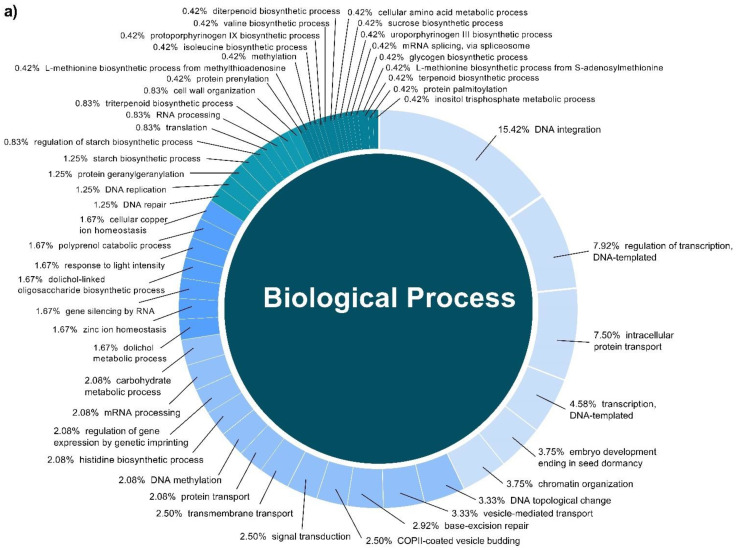

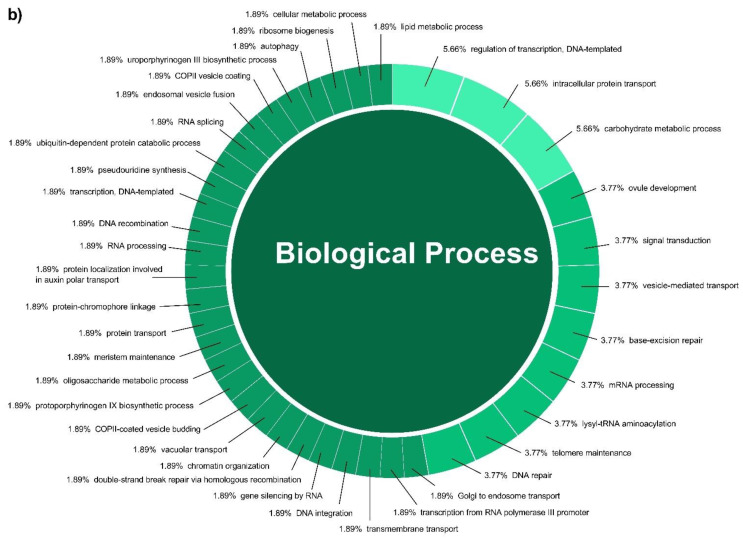
Biological Process categories of the GO analysis of both conserved (**a**) and novel (**b**) *C. cyminum* miRNAs’ putative target genes.

**Figure 5:**
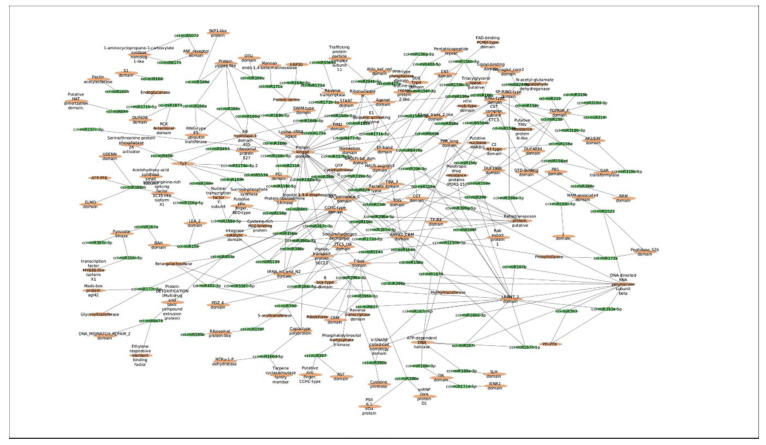
MFE-based network interaction of *C. cyminum* miRNAs and their corresponding potential targets.

In this study, a total of 3037 putative target genes for *C. cyminum* miRNAs (1959 target genes for conserved miRNAs and 1078 target genes for novel miRNAs) were identified, among which cci-miR156b-3p (240) had the highest possible number of targets among conserved miRNAs, followed by cci-miR156d-3p (239) and cci-miR156f-3p (218), whereas cci-miRN10-3p (274) targeted the most number of transcripts among novel miRNAs. The Gene Ontology (GO) analysis showed that the top terms in the Biological Process (BP) category for the targets of conserved miRNAs were “DNA integration” (15.42%), “regulation of transcription, DNA-templated” (7.92%), and “intracellular protein transport” (7.50%); moreover, “histidine biosynthetic process” (2.08%), “dolichol metabolic process” (1.67%), “dolichol-linked oligosaccharides biosynthetic process” (1.67%), and “triterpenoid biosynthetic process” (0.83%) were key terms related to secondary metabolism biosynthesis. While “regulation of transcription, DNA-templated” (5.66%), “intracellular protein transport”, and “carbohydrate metabolic process” (5.66%) were the three main terms for the targets of novel miRNAs in the BP category and, in particular, “protoporphyrinogen IX biosynthetic process” (0.66%) is involved in the biosynthesis of secondary metabolites (Figure 4). Furthermore, gene network analysis revealed the coregulation of numerous target genes (Figure 5).

### 2.4. Identification of C. cyminum miRNA Targets Involved in Plant Secondary Metabolite Biosynthesis

**Figure 6:**
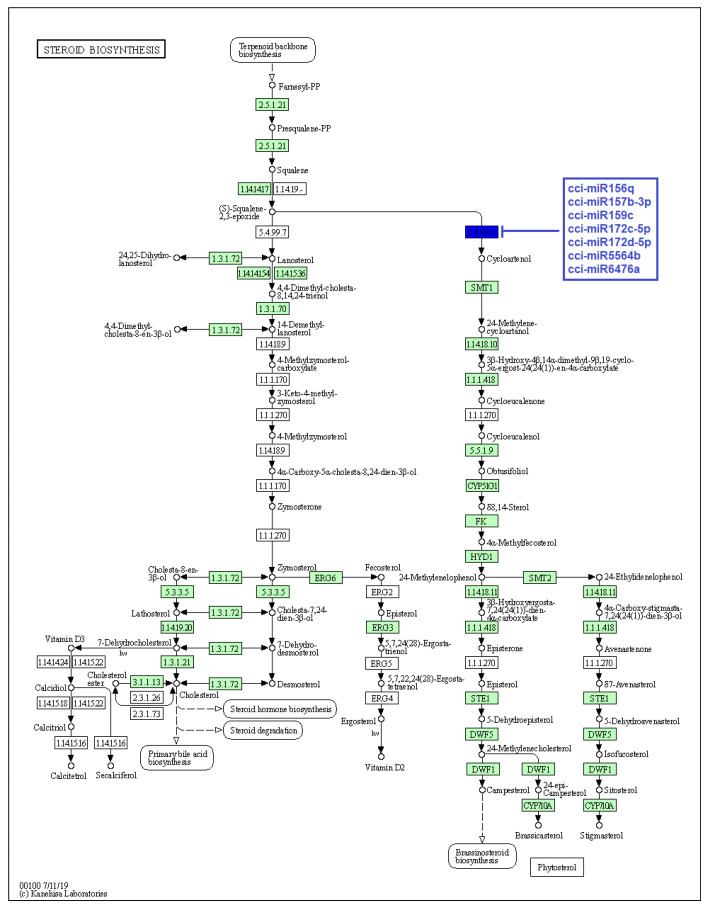
Target enzymes of conserved miRNAs of *C. cyminum* in the steroid biosynthesis via terpenoid backbone biosynthesis pathway. EC:5.4.99.8—Cycloartenol synthase. The blue box represents the targeted enzyme of the corresponding known miRNAs.

**Figure 7:**
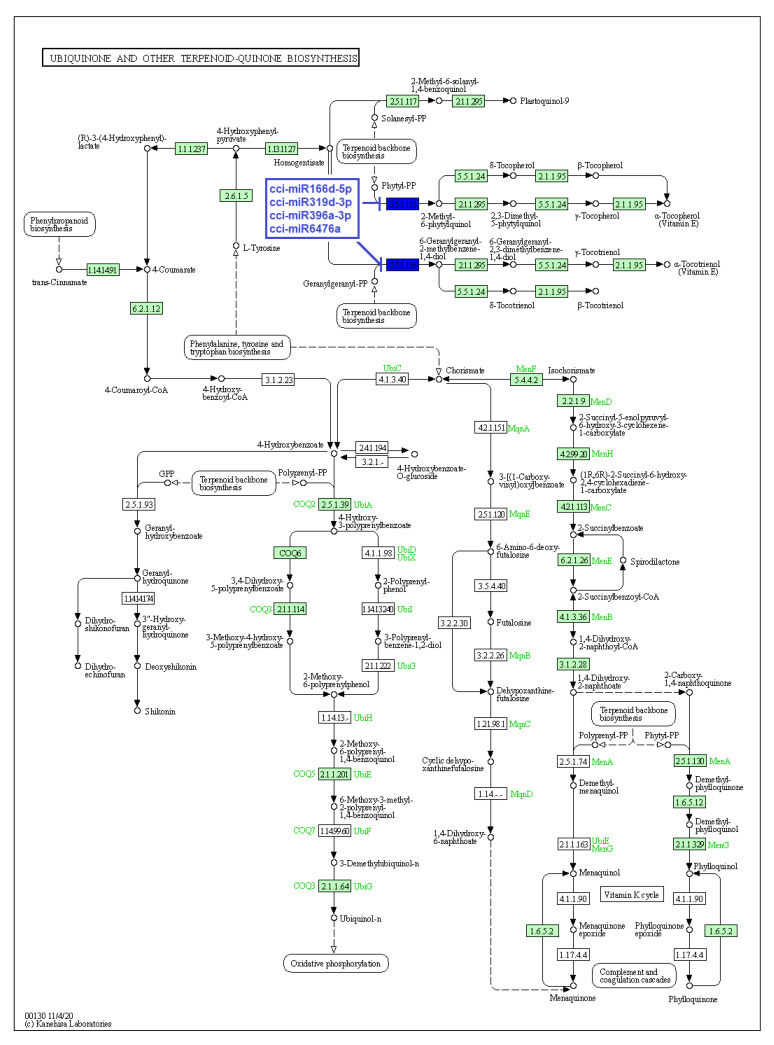
Target enzymes of conserved miRNAs of *C. cyminum* in the ubiquinone and other terpenoid-quinone biosynthesis pathways. EC:2.5.1.115—Homogentisate phytyltransferase; EC: 2.5.1.116—Homogentisate genarylgeranyltransferase. The blue boxes represent the targeted enzymes of the corresponding known miRNAs.

**Figure 8:**
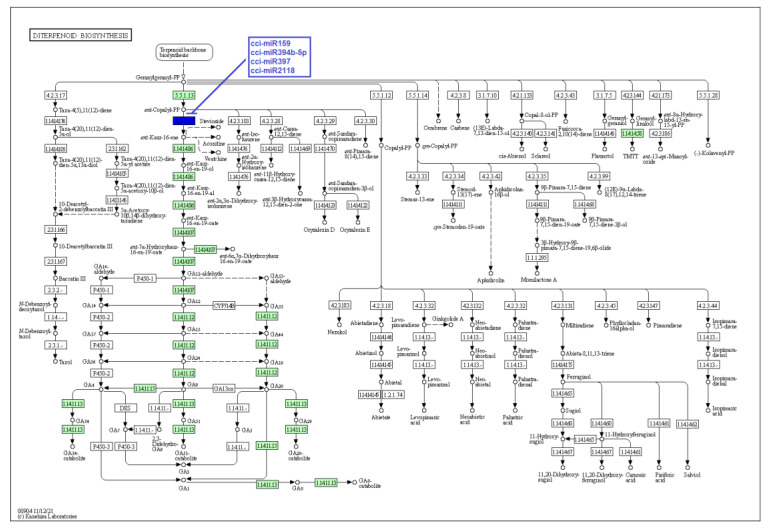
Target enzymes of conserved miRNAs of *C. cyminum* in the diterpenoid biosynthesis pathway. EC:4.2.3.19—Ent-kaurene synthase. The blue box represents the targeted enzyme of the corresponding known miRNAs.

Secondary metabolites produced by particular enzymes are thought to be responsible for *C. cyminum*’s pharmacological activities. Thus, as stated above, the main objective of the study is the identification of *C. cyminum* miRNAs that are involved in the biosynthesis of secondary metabolites as they are associated with target genes that encode the enzymes involved in different metabolic and biosynthesis pathways. Data analysis revealed that a total of 70 *C. cyminum* miRNA target genes are associated with the biosynthesis of steroids, diterpenoids, sesquiterpenoids, triterpenoids, terpenoids, ubiquinones and other terpenoid-quinones; of which fourteen conserved miRNAs (cci-miR156q, cci-miR157b-3p, cci-miR159, cci-miR159c, cci-miR166d-5p, cci-miR172c-5p, cci-miR172d-5p, cci-miR319d-3p, cci-miR394b-5p, cci-miR396a-3p, cci-miR397, cci-miR2118, cci-miR5564b, and cci-miR6476a) denoted control over key terpene-regulating enzymes (Cycloartenol synthase, homogentisate phytyltransferase, homogentisate geranylgeranyltransferase, and ent-kaurene synthase) within the plant metabolism, leading to the biosynthesis of different terpenoids shown in Figure 6, Figure 7 and Figure 8.

**Figure 9:**
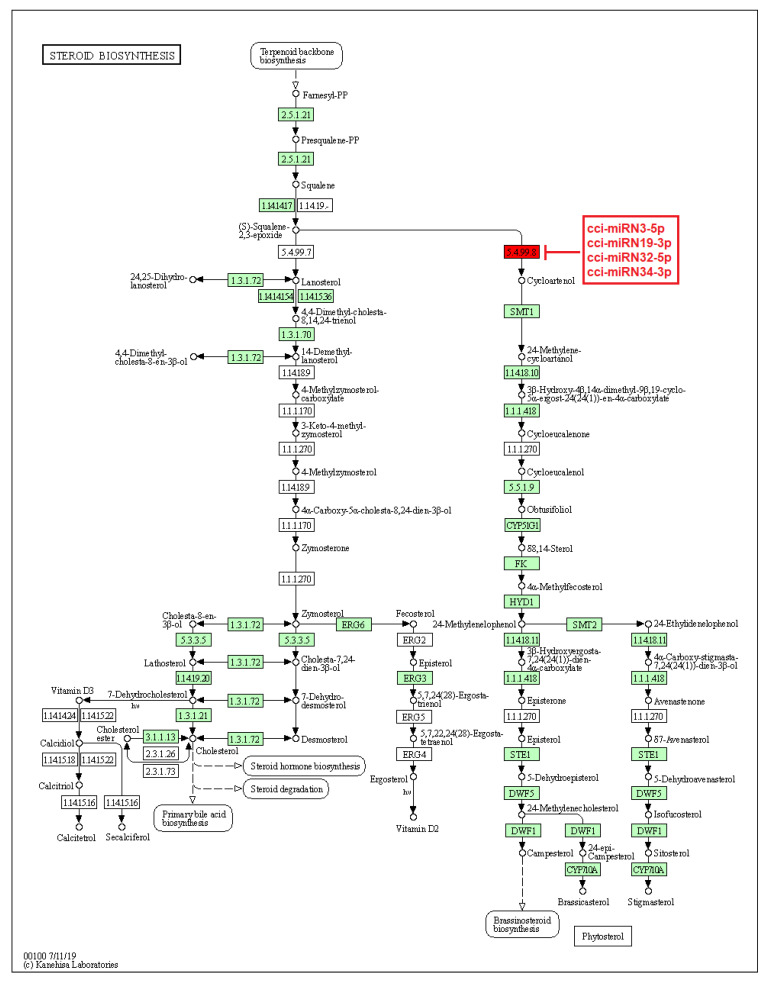
Target enzymes of novel miRNAs of *C. cyminum* in the steroid biosynthesis via terpenoid backbone biosynthesis pathway. EC:5.4.99.8—Cycloartenol synthase. The red box represents the targeted enzyme of the corresponding novel miRNAs.

**Figure 10:**
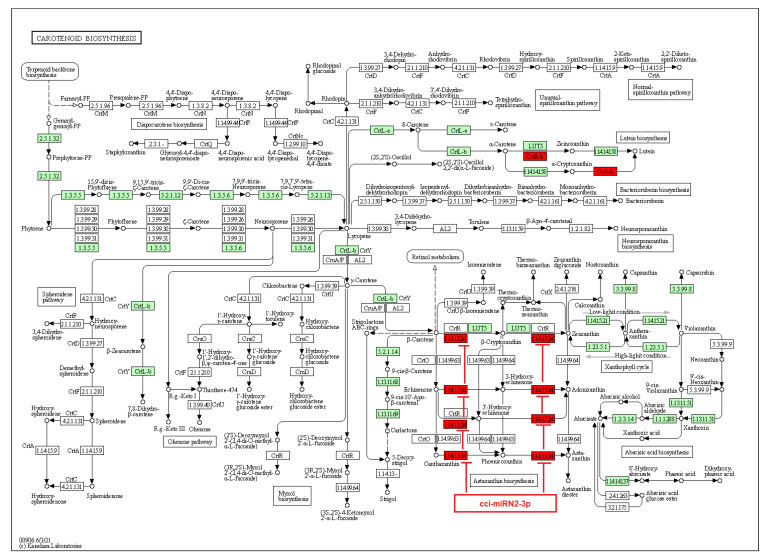
Target enzymes of novel miRNAs of *C. cyminum* in the carotenoid biosynthesis pathway. EC:1.14.15.24—Beta-carotene 3-hydroxylase. The red boxes represent the targeted enzymes of the corresponding novel miRNAs.

On the other hand, a few identified novel *C. cyminum* miRNAs were also found to be associated with secondary metabolite biosynthetic pathways, including the biosynthesis of stilbenoid, diarylheptanoid, gingerol, phenylpropanoid, flavonoids, diterpenoids, terpenoids, sesquiterpenoids, and triterpenoids. Four of the novel miRNAs (cci-miRN3-5p, cci-miRN19-3p, cci-miRN32-5p, and cci-miRN34-3p) were considerably linked with the enzyme cycloartenol synthase within the terpenoid backbone biosynthesis pathway (Figure 9), while novel miRNA cci-miRN2-3p was revealed to be correlated with the enzyme beta-carotene 3-hydroxylase (Figure 10) associated with the production of B-end group carotenoids.

### 2.5. Experimental Validation of C. cyminum miRNAs by qPCR

**Figure 11:**
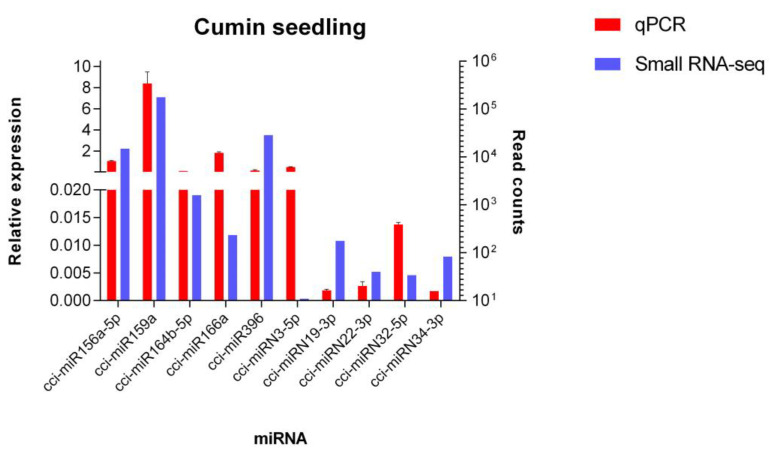
Quantitative PCR analysis of novel and conserved miRNAs from *C. cyminum.* The relative expression of five conserved miRNAs (cci-miR156a-5p, cci-miR159a, cci-miR164b-5p, cci-miR166a, and cci-miR396) and five novel miRNAs (cci-miRN3-5p, cci-miRN19-3p, cci-miRN22-3p, cci-miRN32-5p, and cci-miRN34-3p) was detected by qPCR. U6 was used as an endogenous control. The analysis was performed in triplicate, and the error bars indicate the standard deviations.

In order to validate the data obtained by high-throughput sequencing, a qPCR analysis was performed. Five conserved and five novel *C. cyminum* miRNAs were randomly selected for the experiments. The results showed similar relative expression patterns with respect to the read counts for all miRNAs analyzed (Figure 11).

## 3. Discussion

Secondary metabolites are synthesized by plants to adapt and respond to the environment’s biotic and abiotic stressors [22]. Moreover, these secondary metabolites have multiple applications in a number of industries that aroused commercial interest and a field of study to explore overproduction alternatives [22]. MiRNAs are short non-coding RNAs that control the plant’s secondary metabolism by targeting the mRNA of crucial enzymes involved in the biosynthetic pathways [11]. Therefore, it becomes imperative to identify the miRNAs and their targets in unexplored medicinal plant species. The present study investigated the miRNA profile of cumin (*C. cyminum*) by Illumina small RNA sequencing technology to better understand their involvement in secondary metabolism.

It is well established that most of the plant miRNA families are highly conserved in the plant kingdom, while some others are specific to certain species (novel miRNAs). The degree of conservation of a miRNA indicates its evolutionary status and is closely related to its expression patterns and functions [23]. In this study, the conserved *C. cyminum* miRNAs from young cumin seedlings (15-day-old seedlings) were distributed in 63 families, from which miR156 was the most persistent family with 42 members and 400,512 read counts. Liu et al. (2018) reported that miR156 controls the phase transition in plant development and accumulates during the juvenile stage, decreasing when the plant ages, which is consistent with our results of cumin miRNAs from the seedling stage [23].

The pharmacological potential of *C. cyminum* is attributed to the presence of bioactive compounds such as terpenoids, phenols, and flavonoids [24]. Several studies reported that cuminaldehyde is the most prominent chemical compound in *C. cyminum* seeds, followed by cuminic alcohol, γ-terpinene, p-cymene, and β-pinene [25,26]. Therefore, terpenoids play a vital role in cumin’s secondary metabolism. Terpenoids backbone is produced through successive condensations of isopentenyl diphosphate (IPP) and its isomer, dimethylallyl diphosphate (DMAPP). After different enzymatic steps, the backbone is arranged and modified to give rise to a morphological and functional diversity of terpenoids [27,28]. Specifically, terpenoids can be classified depending on the number of carbons (or isoprenoid units, C5H8) in C10 monoterpenes, C15 sesquiterpenes, C20 diterpenes, C30 triterpenes, and C40 tetraterpenes. Particularly, C30 triterpenoids contain a carbon skeleton of four or five rings of varied configurations, while steroids are tetracyclic compounds produced from terpenoid precursors and contain a perhydro-1,2-cyclopentane-phenanthrene moiety [29]. For both triterpenoids and steroids, a common step is needed in their biosynthesis pathway, which consists of the cyclization of squalene (C30 compound) into cycloartenol by the enzyme cycloartenol synthase [27]. This enzyme belongs to the oxidosqualene cyclase gene family [30]. Interestingly, the present study revealed that cycloartenol synthase is targeted by the conserved cumin miRNAs cci-miR156q, cci-miR157b-3p, cci-miR159c, cci-miR172c-5p, cci-miR172d-5p, cci-miR5564b, and cci-miR6476a and the novel cumin miRNAs cci-miRN3-5p, cci-miRN19-3p, cci-miRN32-5p, and cci-miRN34-5p. It suggests these cumin miRNAs can target a key regulator enzyme in both sterol and triterpenoid production due to squalene cyclization being a crucial branch point in their biosynthesis pathway [30]. Our results coincide with the findings of Srivastava et al. (2018), who reported that miR5140 and miR159 families were involved in the gene regulation of cycloartenol synthase and sterol delta-7 reductase 1, which are directly involved in the biosynthesis of withanolides, a group of steroids produced by the medicinal plant *Withania somnifera* [17]. Similarly, Jeena et al. (2021) identified the cleavage site of cycloartenol synthase by Bm-miR172c-5p from the medicinal plant *Bacopa monnieri* and suggested that this miRNA could be a key regulator in the plant response to abiotic stress [31].

Additionally, our study showed that the conserved cumin miRNAs cci-miR166d-5p, cci-miR319d-3p, cci-miR396a-3p, and cci-miR6476a target both enzymes homogentisate phytyltransferase and homogentisate genarylgeranyltransferase. These enzymes are responsible for condensing homogentisic acid to phytyl-pyrophosphate (Phytyl-PP) and geranylgeranyl pyrophosphate (GGPP) for later production of tocopherols and tocotrienols in the Ubiquinone and Other Terpenoid-Quinone Biosynthetic Pathway. Importantly, these enzymes perform the first committed reaction in their biosynthesis due to an irreversible enzymatic reaction in which the product must continue through the pathway [32]. Our data are consistent with the report of Zheng et al. (2019), in which several miRNAs of soybean (*Glycine max*) targeted genes of the same biosynthetic pathway under biotic stress [33].

Moreover, in this investigation, we showed that the conserved cumin miRNAs cci-miR159, cci-miR394b-5p, cci-miR397, and cci-miR2118 target the enzyme ent-kaurene synthase, responsible for producing ent-Kaur-16-ene from ent-copalyl diphosphate, playing a key bifunctional role in the biosynthesis of phytohormones and terpenoids [34,35]. Additionally, this enzyme has been conserved in all land plants and reported in higher concentrations in rapidly dividing plant tissues [36]. Likewise, Barozai et al. (2011) found in a computational study that the miRNA families miR158 and miR393 from *Picea glauca* and *Picea sitchensis* targeted ent-kaurene synthase [37].

Regarding carotenoids, they are C40 tetraterpenoids that play an important role in the physical condition of plants and are biomolecules of great commercial interest due to their different applications in fragrances, food, cosmetics, and biofuels [38]. In addition, they have antioxidant, anti-inflammatory, and anti-allergic properties [39]. Carotenoids are made by two C20 moieties of GGPP, linked together at their tails to give up a C40 linear hydrocarbon skeleton adaptable to different structural changes [39]. Each GGPP moiety is produced by the linear sequential addition of three IPP molecules to one DMAPP molecule [39,40,41]. We noticed that the novel miRNA cci-miRN2-3p targets the enzyme beta-carotene 3-hydroxylase, which plays a vital role in several sequential steps for the synthesis of astaxanthins. Astaxanthins are carotenoids with high pharmaceutical importance synthesized by ketolase and beta-carotene 3-hydroxylase enzymes from β-carotene [42]. Importantly, beta-carotene 3-hydroxylase and other related enzymes are responsible for rate-limiting steps in β-carotene catalysis [43]. Therefore, the cumin novel miRNA cci-miRN2-3p could represent a key regulator in astaxanthins biosynthesis. Similarly, another study of potato seedling miRNAs revealed that the novel miRNA stu-novel-miR5125 targeted beta-carotene 3-hydroxylase, which could be upregulated by low-temperature stress [44]. Furthermore, a miRNA regulatory network from *Cryptomeria fortunei*, a medicinal plant from China, indicated that several miRNAs, including pde-miR159, mdm-miR396a, and hbr-miR6173 were related to the carotenoid biosynthesis [45].

Finally, the qPCR experiment to validate the sequencing analysis was done with five conserved and five novel *C. cyminum* miRNAs. The results demonstrated that both the Illumina sequencing and the qPCR assays displayed similar expression patterns. This behavior was also observed in the leaves of the medicinal plant *C. camphora* [18], 15-day-old seedlings of wild-type *A. thaliana* [46], and 15-day-old seedlings of Tibetan barley (*Hordeum vulgare* L. var. *nudum*) [47].

## 4. Materials and Methods

### 4.1. Plant Materials, RNA Extraction and Quality Control

*C. cyminum* seeds were collected from a local supermarket and washed thoroughly with 2% sodium hypochlorite and colloidal silver (10 drops of Microdacyn^®^ in 100 mL of distilled water). For seed germination, Petri dishes were prepared with sterile filter paper, 2 mL of MS 1/2 medium, and a thin layer of cumin seeds, which were left incubating at 25 °C for 15 days with a 12-h light/dark photoperiod. Seedling samples of healthy 15-days-old-*C. cyminum* were collected and instantly frozen in liquid nitrogen. Total RNA (including small RNA) was isolated from 100 mg of the seedling samples using the Direct-zol RNA micro kit (Zymo Research, Irvine, CA, USA) following the manufacturer’s instructions. RNA concentration and purity were quantified utilizing a Nanodrop2000 Spectrophotometer (Thermo Scientific, Wilmington, DE, USA). The integrity of the samples was assessed on Tapestation (Agilent, Santa Clara, CA, USA).

### 4.2. Small RNA Library Construction and Sequencing

A single small RNA sequencing library from pooled RNA samples (3 biological replicates) was prepared using QIAseq^®^ miRNA Library Kit (Qiagen, Germantown, MD, USA). Briefly, 63 ng of total RNA was used as starting material, and 3′ adapters were ligated to the specific 3′OH group of miRNAs, followed by ligation of 5′ adapter. Subsequently, the adapter-ligated fragments were reverse transcribed with Unique Molecular Index (UMI) assignment, and the cDNA was enriched and barcoded by PCR amplification. The resultant cDNA library was then quantified by Qubit fluorometer (Thermo Fisher Scientific, Waltham, MA, USA), and the fragment size distribution of the library was evaluated on Agilent 2200 TapeStation system. Finally, high-throughput single-ended sequencing was performed using an Illumina NovaSeq 6000 for 75 cycles, following the manufacturer’s instructions.

### 4.3. Small RNA Sequencing Data Analysis

Illumina GA raw data was processed by srna-workbench (V3.0_ALPHA). For this step, 3′ adaptors and low-quality reads (<Q30) were removed, and reads matching other ncRNAs such as rRNAs, tRNAs, snRNAs, and snoRNAa, as well as sequences smaller than 16 bp and larger than 40 bp were eliminated. The remaining small RNA sequences were aligned against miRbase22 (http://www.mirbase.org, accessed on 10 January 2022) in order to identify conserved *C. cyminum* miRNAs. Those sequences that did not show homology were considered for the prediction of novel miRNAs using bowtie (https://bowtie-bio.sourceforge.net/index.shtml, accessed on 10 January 2022) and Mireap_0.22b [48]. Conserved miRNAs were identified by the homology approach against mature Viridiplantae miRNA sequences due to the unavailability of the *C. cyminum* genome. In this study, only novel miRNAs with suitable precursor secondary structures and with MFEI values ≥ 0.70 were considered. The secondary structures of the precursors were predicted using the UNAFold web server (http://www.unafold.org, evaluated on 16–17 June 2022), and the MFEI values were calculated using the following formula:

$$MFEI=\frac{\left(MFE/length\;of\;RNA\;sequence\right)\times 100}{\%\;GC\;content}$$

### 4.4. Prediction of C. cyminum miRNA Targets, Their Functional Annotation, and Pathway Analysis

The psRNATarget tool (https://www.zhaolab.org/psRNATarget/, accessed on 10 January 2022) was used to predict the target genes of both novel and conserved miRNAs with copy numbers equal to or greater than five. Potential *Cuminum cyminum* miRNA targets were annotated using the Gene Ontology (GO) in the bioprocesses (BP) domain. Additionally, a biological network of the miRNAs and their targets was built using the MFE values of the miRNA-target interaction and visualized using Cytoscape 3.2 (https://cytoscape.org/releasenotes320.html, accessed on 10 January 2022) in order to identify the coregulation of possible targets. Finally, a thorough analysis of miRNA targets linked to pathways for the biosynthesis of secondary metabolites was performed.

### 4.5. Extraction of Small RNA and Experimental Validation of C. cyminum miRNAs by qPCR

In order to validate the identified conserved and novel miRNAs, small RNA was extracted from frozen *C. cyminum* seedlings using a mini miRNeasy kit (Qiagen, Maryland, USA) following the manufacturer’s instructions. Quality and quantity of small RNA samples were measured using NanoDrop One UV–Vis microvolume spectrophotometer (Thermo Scientific, Wilmington, DE, USA). Resulting small RNAs were then polyadenylated and reverse transcribed using the Mir-X miRNA First-Strand Synthesis kit (Clontech, Mountain View, CA, USA), and finally, the qPCR was performed using the TB Green^®^ Advantage^®^ qPCR Premix (Takara Bio USA, Inc., San José, CA, USA) in a StepOne™ Real-Time PCR System (Applied Biosystems, Carlsbad, CA, USA). The primers for cumin novel miRNAs were listed in Supplementary Table S2. The reactions were performed in a 48-well optical plate with the following conditions: initial polymerase activation step for 10 s at 95 °C, denaturation by 45 cycles of 5 s at 95 °C, annealing and extension for 20 s at 55 °C. Following the amplification cycle, a melting curve analysis with temperature ranges of 56–95 °C and increments of 0.5 °C every 10 s was performed. For each sample, the reactions were run with three technical and six biological replicates, and the relative expression of the miRNAs was normalized to U6, the endogenous control.

## 5. Conclusions

In this study, a total of 353 conserved and 39 novel cumin miRNAs were identified using Illumina high-throughput sequencing technology. Among the identified miRNAs, fourteen conserved miRNAs (cci-miR156q, cci-miR157b-3p, cci-miR159, cci-miR159c, cci-miR166d-5p, cci-miR172c-5p, cci-miR172d-5p, cci-miR319d-3p, cci-miR394b-5p, cci-miR396a-3p, cci-miR397, cci-miR2118, cci-miR5564b, and cci-miR6476a) and four novel miRNAs (cci-miRN3-5p, cci-miRN19-3p, cci-miRN32-5p, and cci-miRN34-3p) were found to target enzymes that were significantly involved in the terpenoid backbone biosynthesis pathway, while one novel miRNA was discovered to target the beta-carotene 3-hydroxylase enzyme connected to the carotenoid biosynthesis pathway. A total of ten random miRNAs (cci-miR156a-5p, cci-miR159a, cci-miR164b-5p, cci-miR166a, cci-miR396, cci-miRN3-5p, cci-miRN19-3p, cci-miRN22-3p, cci-miRN32-5p, and cci-miRN34-3p) were experimentally verified using qPCR. To the best of our knowledge, this is the first report of microRNA profile from the culinary and medicinal plant *C. cyminum* and its relationship with the biosynthesis of secondary metabolites. This study could support miRNA-mediated transgenic research for the overproduction of valuable plant secondary metabolites for both medical and commercial purposes.

## Figures and Tables

**Table 1:** Categorization of sequencing reads (srna-workbench, Bowtie, Mireap_0.22b, and UNAFold bioinformatic tools were used during the analysis).

Category	Total Reads	Unique Reads
Total Reads	17,058,681	10,956,054
Trimmed Reads	12,917,048	2,126,409
% reads aligned to ncRNA	21.16%	
Reads aligned to ncRNA (rRNA, snoRNA, snRNA, tRNA)	7,342,240	582,748
Reads aligned to mirBase	935,185	866,348
Known miRNA	353	
Reads utilized for novel miRNA	1,057,102	232,425
Novel miRNA	39	
Putative reads	3,582,521	866,078

**Table 2:** Summary of conserved miRNAs in *C. cyminum*.

miRNA Family	Name	Sequence (5′–3′)	Length (nt)	Reference miRNA	No. of Mismatches	Read Counts	E Value
MIR156	cci-miR156d-3p	GCTCTCTATGCTTCTGTCATCA	22	stu-miR156d-3p	0	213,619	0.00000009
	cci-miR156	TTGACAGAAGATAGAGAGCAC	21	bgy-miR156	0	155,965	0.0000004
	cci-miR156a	TGACAGAAGAGAGTGAGCACA	21	bna-miR156a	0	14,927	0.0000004
MIR159	cci-miR159a	TTTGGATTGAAGGGAGCTCTA	21	ath-miR159a	0	177,734	0.0000004
	cci-miR319b	TTGGACTGAAGGGAGCTCCCT	21	ath-miR319b	0	5257	0.0000003
	cci-miR319d-3p	CTTGGACTGAAGGGAGCTCCC	21	ppt-miR319d-3p	0	346	0.0000003
MIR160	cci-miR160h	TGCCTGGCTCCCTGCATGCCA	21	ptc-miR160h	0	149	0.0000003
	cci-miR160a-3p	GCGTATGAGGAGCCAAGCATA	21	gma-miR160a-3p	0	7	0.0000002
	cci-miR160d	TGCCTGGCTCCCTGAATGCCA	21	cpa-miR160d	0	1	0.0000002
MIR164	cci-miR164b-5p	TGGAGAAGCAGGGCACGTGCA	21	ath-miR164b-5p	0	1596	0.0000003
	cci-miR164d	TGGAGAAGCAGGGCACATGCT	21	mtr-miR164d	0	116	0.0000002
	cci-miR164g-3p	CACGTGCTCCCCTTCTCCA	19	zma-miR164g-3p	0	36	0.000002
MIR164_2	cci-miR164b	TGGAGAAGCAGGGCACTT	18	far-miR164b	0	3	0.000009
MIR166	cci-miR166c	TCGGACCAGGCTTCATTCCTC	21	mtr-miR166c	0	140,263	0.0000004
	cci-miR166u	TCTCGGACCAGGCTTCATT	19	gma-miR166u	0	2404	0.000005
	cci-miR166c-5p	GGAATGTTGTCTGGCTCGAGG	21	gma-miR166c-5p	0	1176	0.0000004
MIR167	cci-miR167h	TTGAAGCTGCCAGCATGA	18	gma-miR167h	0	9	0.000009
MIR167_1	cci-miR167d	TGAAGCTGCCAGCATGATCTGG	22	ath-miR167d	0	21,213	0.00000009
	cci-miR167c-5p	TGAAGCTGCCAGCATGATCTGC	22	tae-miR167c-5p	0	2262	0.00000008
	cci-miR167d	TGAAGCTGCCAGCATGATCTGA	22	cpa-miR167d	0	1244	0.00000007
MIR168	cci-miR168c-5p	TCGCTTGGTGCAGGTCGGGAC	21	bra-miR168c-5p	0	694	0.0000004
	cci-miR168	TCCCGCCTTGCATCAATTGAAT	22	aau-miR168	1	13	0.000009
	cci-miR168a-5p	TCGCTTGGTGCAGATCGGGAC	21	osa-miR168a-5p	0	13	0.0000002
MIR169_1	cci-miR169k	TAGCCAAGGATGACTTGCCTGC	22	bna-miR169k	0	232	0.00000008
	cci-miR169n	TAGCCAAGAATGACTTGCCT	20	osa-miR169n	0	52	0.0000006
	cci-miR169h	TAGCCAAGGATGACTTGCCTG	21	ath-miR169h	0	3	0.0000002
MIR169_2	cci-miR169a-5p	CAGCCAAGGATGACTTGCCGA	21	ath-miR169a-5p	0	91	0.0000001
	cci-miR169r-3p	GGCAAGTTGTCTTTGGCTACA	21	zma-miR169r-3p	1	45	0.00004
	cci-miR169a-3p	GGCAAGTTGTCTTTGGCTAC	20	ath-miR169a-3p	1	11	0.0001
MIR171_1	cci-miR171b-3p	TTGAGCCGTGCCAATATCACG	21	ath-miR171b-3p	0	879	0.0000003
	cci-miR171d-5p	TTGGCCGGGCTCACTCAGA	19	osa-miR171d-5p	1	470	0.0007
	cci-miR171d	TGATTGAGCCGTGCCAATATC	21	cpa-miR171d	0	172	0.0000003
MIR172	cci-miR172c-5p	GTAGCATCATCAAGATTCACA	21	mtr-miR172c-5p	0	217	0.0000004
	cci-miR172d-5p	AGCACCATCAAGATTCACA	19	zma-miR172d-5p	0	174	0.000002
	cci-miR172a	AGAATCTTGATGATGCTGCAT	21	ath-miR172a	0	38	0.0000002
MIR319	cci-miR319i	TTGGGCTGAAGGGAGCTCCC	20	ptc-miR319i	0	7	0.0000006
MIR390	cci-miR390b	AAGCTCAGGAGGGATAGCGCC	21	ppt-miR390b	0	196	0.0000003
	cci-miR390a-3p	CGCTATCTATCCTGAGTTTCA	21	ath-miR390a-3p	1	63	0.00005
	cci-miR390	AAGCACAGGATGGATAGCG	19	pta-miR390	1	14	0.0006
MIR393	cci-miR393a-5p	TCCAAAGGGATCGCATTGATCC	22	ath-miR393a-5p	0	66	0.00000007
	cci-miR393	TCCAAAGGGATCGCATTGAT	20	ghr-miR393	0	23	0.0000006
	cci-miR393c-3p	ATCATGCTATCCCTTTGGATT	21	gma-miR393c-3p	0	19	0.0000003
MIR3932	cci-miR3932a	TCGTCGTCATCACAAAGTT	19	ath-miR3932a	1	1	0.0006
MIR394	cci-miR394b-5p	TTGGCATTCTGTCCACCTCC	20	ath-miR394b-5p	0	119	0.0000006
MIR395	cci-miR395b	CTGAAGTGTTTGGGGGAACTCC	22	sly-miR395b	0	821	0.00000004
	cci-miR395o	GAGTTCCTCCAAACACTT	18	osa-miR395o	0	3	0.000009
	cci-miR395a	CTGAAGTGTTCGGGGGAACTC	21	ath-miR395a	1	2	0.00004
MIR396	cci-miR396f	TTCCACGGCTTTCTTGAACTG	21	ptc-miR396f	0	41,200	0.0000004
	cci-miR396	TTCCACAGCTTTCTTGAACTT	21	pta-miR396	0	28,712	0.0000004
	cci-miR396a-3p	GTTCAATAAAGCTGTGGGAAG	21	ath-miR396a-3p	0	6661	0.0000003
MIR397	cci-miR397	ATTGAGTGCAGCGTTGATGA	20	lja-miR397	0	21	0.0000006
MIR398	cci-miR398a-3p	TGTGTTCTCAGGTCACCCCTT	21	ath-miR398a-3p	0	85	0.0000002
	cci-miR398c	TGTGTTCTCAGGTCGCCCCTG	21	gma-miR398c	0	81	0.0000001
	cci-miR398a-5p	GGAGTGTCATGGGAACACA	19	aly-miR398a-5p	1	4	0.0006
MIR399	cci-miR399i	TGCCAAAGGAGAATTGCCCTG	21	gma-miR399i	0	15	0.0000002
	cci-miR399i	TGCCAAAGGAGAGTTGCCCTA	21	ptc-miR399i	0	15	0.0000002
	cci-miR399d	TGCCAAAGGAGATTTGCCCCG	21	ath-miR399d	0	1	0.0000002
MIR403	cci-miR403-3p	TTAGATTCACGCACAAACTCG	21	ath-miR403-3p	0	4500	0.0000003
	cci-miR403a	TTAGATTCACGCACAAACTT	20	gma-miR403a	0	471	0.0000009
	cci-miR403-5p	TTAGATTCACGCACAAAA	18	bra-miR403-5p	0	7	0.00001
MIR408	cci-miR408	TGCACTGCCTCTTCCCTGG	19	cpa-miR408	0	35	0.000003
MIR414	cci-miR414	TGACGATGATGATGATGATG	20	ath-miR414	1	1	0.0002
MIR437	cci-miR437x-5p	GTTTGACTTAGGACAACTCTA	21	sbi-miR437x-5p	0	2	0.0000002
MIR444	cci-miR444b.2	TGCAGTTGTTGTCTCAAGCTT	21	osa-miR444b.2	0	2	0.0000002
	cci-miR444b	CTTGAGACAGCAACTGCA	18	hvu-miR444b	0	1	0.00001
	cci-miR444d.3	TTGTGGCTTTCTTGCAAGTTG	21	osa-miR444d.3	0	1	0.0000002
MIR477	cci-miR477-5p	ACTCTCCCTCAAAGGCTTC	19	ppe-miR477-5p	0	455	0.000005
	cci-miR477a	ACTCTCCCTCAAGGGCTTCTG	21	nta-miR477a	0	265	0.0000003
	cci-miR477a-5p	TCTCCCTCAGAGGCTTCC	18	ptc-miR477a-5p	0	1	0.000009
MIR482	cci-miR2118	TTTCCTATTCCACCCATCCCAT	22	pgi-miR2118	0	43	0.00000004
MIR529	cci-miR529-5p	AGAAGAGAGAGAGTACAGCCT	21	zma-miR529-5p	0	1	0.0000002
MIR774	cci-miR774b-5p	GTCATCCAAACCTTCATCT	19	aly-miR774b-5p	1	1	0.0006
MIR818	cci-miR1130b-3p	TTATATTAAGGGACGGAGG	19	tae-miR1130b-3p	1	15	0.0007
	cci-miR1436	ACATTATGAGACGGAGGGAGT	21	osa-miR1436	1	2	0.00004
	cci-miR1439	TTTTGGGACGGAGTGAGTA	19	osa-miR1439	1	1	0.0006
MIR821	cci-miR821d	CAACTTTGTTGTTGTTGAC	19	sbi-miR821d	1	1	0.0009
	cci-miR821e	AAGTCATCAAAACAAAAGT	19	sbi-miR821e	1	1	0.0006
MIR827	cci-miR827	TTAGATGATCATCAGCAAACA	21	osa-miR827	1	5537	0.00007
	cci-miR827-5p	TTTGTTGGTGGTCATCTAA	19	bdi-miR827-5p	1	251	0.0007
	cci-miR827	TTAGATGAACATCAGCAAACA	21	nta-miR827	1	3	0.00004
MIR828	cci-miR828	TCTTGCTTAAATGAGTGTTCCA	22	ath-miR828	1	24	0.000009
MIR834	cci-miR834	TGGTAGCAGTAGTGGTGGT	19	ath-miR834	1	2	0.0006
MIR835	cci-miR835-5p	TTCTTGCATATGTTCTTT	18	ath-miR835-5p	0	1	0.000009
MIR845_3	cci-miR845b	CAATTGGTATCAGAGCTA	18	vvi-miR845b	0	2	0.00001
MIR858	cci-miR858a	TTTCGTTGTCTGTTCGACCTT	21	ath-miR858a	0	1090	0.0000002
	cci-miR858b	TTCGCTGTCTGTTCGACCTTG	21	ath-miR858b	1	3	0.00004
	cci-miR858-3p	TTCGTTGTCTGCTCGACC	18	aly-miR858-3p	0	2	0.000009
MIR862	cci-miR862-3p	ATATGCTGGATTTACTTGAAG	21	ath-miR862-3p	1	1	0.00004
MIR1120	cci-miR1120a	TTATATTATGAGACGGAG	18	tae-miR1120a	0	5	0.00001
MIR1122	cci-miR1133	GGACGGAGGGAGTATATG	18	tae-miR1133	0	3	0.00002
	cci-miR5281e	ATAAATAGAACCGGAGGGAG	20	mtr-miR5281e	1	2	0.0001
MIR1511	cci-miR1511	ACCTAGCTCTGATACCATGA	20	mdm-miR1511	0	2	0.0000006
MIR1520	cci-miR1520q	ACCAATTAGAACATGACACA	20	gma-miR1520q	1	1	0.0002
MIR1863	cci-miR1863b	AGCTCTGATACCATATTAACTG	22	osa-miR1863b	1	4	0.00001
MIR2084	cci-miR2084	CCTGCATTGGTGGATTGTG	19	ppt-miR2084	1	1	0.0006
MIR2275	cci-miR2275b-3p	AGATATTAGAGAAAACTGA	19	zma-miR2275b-3p	1	3	0.0007
MIR2275	cci-miR2275d-5p	AGAGTTGGAGTAAAGAAAA	19	zma-miR2275d-5p	1	1	0.0006
MIR2646	cci-miR2646b	ATGACATGTAGTGATGATGT	20	mtr-miR2646b	1	1	0.0002
MIR2673	cci-miR2673b	CCTCTTCCTCTTCCTCTTCC	20	mtr-miR2673b	0	2	0.0000009
MIR2912	cci-miR2912a	TCTAGAACTCCAGATATGG	19	peu-miR2912a	1	3	0.0006
MIR3630	cci-miR3630-3p	TGGGAATCTCTTTGATGCAC	20	vvi-miR3630-3p	1	3	0.0003
MIR5067	cci-miR5181-3p	CACTTATTTTGGAACGGAGGG	21	ata-miR5181-3p	1	4	0.00004
	cci-miR5049d	ACAACTATTTAGGAACGGAG	20	hvu-miR5049d	1	3	0.0002
	cci-miR5181-5p	GACAATTATTCTGGATCGG	19	ata-miR5181-5p	1	1	0.0006
MIR5298	cci-miR5298a	TTCTTCATCTTCATCTCAT	19	mtr-miR5298a	1	1	0.0006
MIR5387	cci-miR5387b	CTTTAGCACCGGCCAGAGCCAC	22	sbi-miR5387b	1	1	0.00001
MIR5564	cci-miR5564a	TGGGGAAGCAATTCGTCGAACA	22	sbi-miR5564a	0	23	0.00000004
	cci-miR5564b	GCAATTCGTCGAACAGCTTG	20	sbi-miR5564b	0	15	0.0000006
MIR5565	cci-miR5565b	TCGCATCAATCCACATGTGTT	21	sbi-miR5565b	1	1	0.00004
MIR5568	cci-miR5568d-5p	TGGCTTTTCTAGACACATAGC	21	sbi-miR5568d-5p	1	1	0.00004
MIR6161	cci-miR6161b	TGGACCAGTATACTTTGCT	19	nta-miR6161b	1	1	0.0006
MIR6476	cci-miR6476a	TCAGTGGAGATGAAACATG	19	ptc-miR6476a	0	97	0.000005
MIR7982	cci-miR7982a	TGGAGGATAATAATATATA	19	stu-miR7982a	1	1	0.0006
MIR7996	cci-miR7996b	TGGTATATATGAAATTTGAA	20	stu-miR7996b	1	1	0.0002
MIR8762	cci-miR8762c	CAACAAAGTTAGCAAACGT	19	gra-miR8762c	1	2	0.0005
NA	cci-miR6478	CCGACCTTAGCTCAGTTGGT	20	ptc-miR6478	0	5673	0.000001
	cci-miR6300	GTCGTTGTAGTATAGTGG	18	gma-miR6300	0	4672	0.00002
	cci-miR894	CGTTTCACGTCGGGTTCACC	20	ppt-miR894	0	744	0.000001

**Table 3:** Potential novel miRNA candidates identified from *C. cyminum*.

Name	Sequence (5′–3′)	Length	Read Count	Strand	MFEI of Precursor
cci-miRN1-3p	UGCUCACUCUCUAUCUGUCACC	22	8	+	1.41
cci-miRN2-3p	GGCGUACCGUGAGCCAAGCAUGC	23	975	−	0.77
cci-miRN3-5p	UAGAAGUGUGACCUGUCUUGCAU	23	11	−	0.90
cci-miRN4-3p	GCAAGGCAGGACACCUUCUAUG	22	194	−	0.98
cci-miRN5-5p	CAGAAGUGUGACCUGUCUUGCAU	23	83	−	0.98
cci-miRN6-5p	UGACAGAAGAGAGUGAGCACA	21	6	−	0.98
cci-miRN7-5p	UGACAGAAGAGAGUGAGCACA	21	6	−	1.08
cci-miRN8-5p	AUUUUAGCUGAAGUAUUUAGUAU	23	5	+	0.75
cci-miRN9-3p	UAGGCAGGCAUUUUUGGCUAGC	22	6	+	0.94
cci-miRN10-3p	UUUAUGGUGAUUUAUUUGUGUGG	23	1382	−	1.18
cci-miRN11-5p	AAACAAAUUCAUCACCAUAAGGA	23	10	−	1.18
cci-miRN12-3p	UUAUGGUGAUUUAUUUGUGUGGG	23	20	−	1.04
cci-miRN13-3p	GAGUGAAUGAAGCGGGAGACUUAU	24	2	−	0.99
cci-miRN14-5p	UAGCUGCUGACUCAUUCAUCCAA	23	12	−	0.99
cci-miRN15-3p	UCAUCUACGCUGCACUCAAUCAU	23	224	−	0.95
cci-miRN16-5p	GGAAUGUUGUCCGGCUCGAUGCU	23	5	−	1.01
cci-miRN17-3p	CUGAUGCAUGAUGUGAGAGCAA	22	6	−	0.90
cci-miRN18-5p	GCUGUCAUCUCAUGCAUUUGGU	22	24	−	0.90
cci-miRN19-3p	CUGAGCCGAACCAAUAUUACUC	22	177	+	0.92
cci-miRN20-5p	GCGUAAUAUUGCUCCGGCUCAGC	23	262	+	0.92
cci-miRN21-5p	AUUUGGCAUUCUGUUCACCUCCA	23	35	+	1.05
cci-miRN22-3p	UGGUGCCACACUGCUCGCGUUU	22	40	−	1.00
cci-miRN23-3p	GCGCUAUCUAUUCUGAGUUUCA	22	134	+	1.00
cci-miRN24-5p	UAGCUGCCGACUCAUUCACUCA	22	8	+	0.89
cci-miRN25-3p	UUUAGUUUUCUCCAAUUUCUCAU	23	67	−	0.81
cci-miRN26-3p	CUUAGUUUUCUCCGAUAUCUCAU	23	10	−	1.12
cci-miRN27-3p	GCAUGUGCUCUUGCUCUCCUGCU	23	52	+	1.12
cci-miRN28-5p	GCAUGCUCCCUUUUUUAUUGGC	22	6	−	0.85
cci-miRN29-3p	CAAGAUACUCUGAAGAAGCUAGC	23	11	−	0.81
cci-miRN30-5p	AUAUUGGCCGGGCUCACUCAGA	22	6	+	0.93
cci-miRN31-3p	UUAACUAUGCGGUCAAAACUCUU	23	5	−	0.80
cci-miRN32-5p	GUUCCCUUGACCACUUCAUUGG	22	34	−	1.10
cci-miRN33-3p	UCGAACGAUGCAGGGGUUGUGUU	23	110	−	0.92
cci-miRN34-3p	UACAUUGAGGGAAAUUGAGGGA	22	83	+	1.17
cci-miRN35-3p	AUCGGACCAGGCUUCAUUCCUC	22	3	−	0.98
cci-miRN36-5p	GGGAUGUUGGCUGGCUCGAUGC	22	7	−	0.98
cci-miRN37-3p	CAAUGUUUCAUUGCGGGUGUGA	22	8	−	0.98
cci-miRN38-5p	CCUCAAUUUCCCUCAGUGUAGU	22	35	−	1.14
cci-miRN39-5p	UGCUUUGUAUAUUUGGAUUUGAU	23	5	−	0.70

## Data Availability

Publicly available datasets were analyzed in this study. This data can be found here: https://www.ncbi.nlm.nih.gov/search/all/?term=SRP376517.

## Supplementary Materials

The following supporting information can be downloaded at: https://www.mdpi.com/article/10.3390/plants12091756/s1, Table S1: Conserved miRNAs in *Cuminum cyminum*, Table S2: Sequence of primers of *C. cyminum* novel miRNAs.
