# Supplementary material for: Identification of Cumin (Cuminum cyminum) MicroRNAs through Deep Sequencing and Their Impact on Plant Secondary Metabolism
Source: Plants (Basel). 2023 Apr 25;12(9):1756. doi: 10.3390/plants12091756 (PMC10180537; doi:10.3390/plants12091756)
Supplement: Supplementary file 1 [file plants-12-01756-s001.zip › plants-2284968-supplementary.pdf]

plants

Table S1: Conserved miRNAs in *Cuminum cyminum*.

miRNA Family	Name	Sequence (5′-3′)	Length (nt)	Reference miRNA	No. of Mismatches	Read Counts	E Value
MIR156	cci-miR156d-3p	GCTCTCTATGCTTCTGTCATCA	22	stu-miR156d-3p	0	213619	0.00000009
	cci-miR156	TTGACAGAAGATAGAGAGCAC	21	bgy-miR156	0	155965	0.0000004
	cci-miR156a	TGACAGAAGAGAGTGAGCACA	21	bna-miR156a	0	14927	0.0000004
	cci-miR156b-3p	TGCTCACTCTCTCTCTGTCACC	22	mtr-miR156b-3p	1	12938	0.00002
	cci-miR156b-3p	GCTCACCCTCTATCTGTCA	19	zma-miR156b-3p	0	1876	0.000005
	cci-miR156g-5p	TTGACAGAAGATAGAGGGCAC	21	mtr-miR156g-5p	0	239	0.0000001
	cci-miR156p	TTGACAGAAGATAGGGAGCAC	21	gma-miR156p	1	200	0.00003
	cci-miR157d-3p	GCTCTCTATGCTTCTGTCATC	21	aly-miR157d-3p	0	166	0.0000003
	cci-miR156l	TGACAGAAGATGGAGAGCAC	20	ptc-miR156l	0	104	0.0000006
	cci-miR157c-3p	GCTCTCTATACTTCTGTCA	19	ath-miR157c-3p	0	97	0.000002
	cci-miR156w	TTGACAGAAGACAGAGAGCAC	21	mdm-miR156w	1	61	0.00004
	cci-miR156	CTGACAGAAGAGAGTGAGCAC	21	ama-miR156	0	49	0.0000004
	cci-miR156i-3p	TGCTCACTTCTCTTTCTGTCA	21	mtr-miR156i-3p	0	41	0.0000002
	cci-miR156e	TGACAGAGGAGAGTGAGCAC	20	vvi-miR156e	0	36	0.0000006
	cci-miR157b-3p	GCTCTCTAAGCTTCTGTCATCA	22	aly-miR157b-3p	0	34	0.00000004
	cci-miR156c	TTGACAGAAGAAAGAGAGCAC	21	smo-miR156c	0	26	0.0000001
	cci-miR156q	TGACAGGAGAGAGTGAGCACT	21	gma-miR156q	1	20	0.00004
	cci-miR156j	TGACAGAAGAGGGTGAGCAC	20	mtr-miR156j	0	17	0.0000006
	cci-miR156e	TGACAGAAGAGAGCGAGCAC	20	sbi-miR156e	0	15	0.0000006
	cci-miR156f-3p	GCTCACTCTCTCTCCGTCACC	21	ath-miR156f-3p	1	11	0.00004
	cci-miR156a	CGACAGAAGAGAGTGAGCAC	20	smo-miR156a	0	11	0.0000006
	cci-miR156ad	TGACAGAAGAAAGTGAGCAC	20	mdm-miR156ad	0	9	0.0000006
	cci-miR156g-3p	GCTCACACTCTCTCTGTCA	19	bdi-miR156g-3p	1	8	0.0006
	cci-miR156b	TGACAGAAGAGAGTGAGCAT	20	cca-miR156b	0	7	0.0000006
	cci-miR156d-3p	GCTCACTCTCTCTTTGTCA	19	ath-miR156d-3p	1	5	0.0006
	cci-miR156a-3p	GCTTACTCTCTCTCTGTCACC	21	bra-miR156a-3p	0	4	0.0000002
	cci-miR156f	TGACAGAAGAGAATGAGCAC	20	nta-miR156f	0	4	0.0000006
	cci-miR156d-3p	GCTCACTCTCTTTCTGTCA	19	aly-miR156d-3p	0	3	0.000002
	cci-miR156a	TGACAGAAGAGAGTGAGTAC	20	hci-miR156a	0	3	0.0000006
	cci-miR156d-3p	GCTCACTCCTCTTTCTGTCA	20	ata-miR156d-3p	0	2	0.0000006
	cci-miR156b-3p	GCTCACTTCTCTCTCTGTCACC	22	bdi-miR156b-3p	0	2	0.00000004
	cci-miR156d-3p	GCTCACTTCTCTGTCTGTCACC	22	bdi-miR156d-3p	1	2	0.000009
	cci-miR156h-3p	GCTCACTTCTCTTCCTGTCA	20	bdi-miR156h-3p	1	2	0.0001
	cci-miR156b-3p	GCTCACTAAGCTTCTGTCATC	21	ahy-miR156b-3p	1	1	0.00004
	cci-miR156b-3p	GCTCACCTCTCTTTCTGTCA	20	aly-miR156b-3p	0	1	0.0000006
	cci-miR156j	TTGACAGAAGAGAGTGAGCAC	21	cme-miR156j	0	1	0.0000003
	cci-miR156d-3p	GCTCACTTATCTTTCTGTCA	20	mtr-miR156d-3p	1	1	0.0001
	cci-miR156k	TGACAGAAGAGAGGGAGCAC	20	ptc-miR156k	0	1	0.0000006
	cci-miR156d-3p	GCTCACTTCTCTATCTGTCACC	22	sly-miR156d-3p	1	1	0.000009
	cci-miR156e-5p	TGATAGAAGAGAGTAAGCAC	20	sly-miR156e-5p	1	1	0.0001
	cci-miR156a-3p	GCTCACTTCTCTCTCTGTCAG	21	zma-miR156a-3p	0	1	0.0000001
	cci-miR156k-3p	GCTCGCTTCTCTTTCTGTCA	20	zma-miR156k-3p	0	1	0.0000006

MIR159	cci-miR159a	TTTGGATTGAAGGGAGCTCTA	21	ath-miR159a	0	177734	0.0000004
	cci-miR319b	TTGGACTGAAGGGAGCTCCCT	21	ath-miR319b	0	5257	0.0000003
	cci-miR319d-3p	CTTGGACTGAAGGGAGCTCCC	21	ppt-miR319d-3p	0	346	0.0000003
	cci-miR159f	TTGGATTGAAGGGGGCTCTA	20	osa-miR159f	1	229	0.0001
	cci-miR319b-5p	AGAGCTTTCTTCGGTCCACT	20	aly-miR319b-5p	0	173	0.0000006
	cci-miR159b-3p	TTTGGGTTGAAGGGAGCTCTT	21	ath-miR159b-3p	1	147	0.00003
	cci-miR319	ATTGGACTGAAGGGAGCTCC	20	cpa-miR319	0	109	0.0000006
	cci-miR159c-5p	TTGGATCGAAGGGAGCTC	18	zma-miR159c-5p	0	100	0.000009
	cci-miR159	TTTGGACTGAAGGGAGCTCTA	21	aqc-miR159	0	92	0.0000002
	cci-miR159b	TTGGATTGAAGAGAGCTC	18	pta-miR159b	0	66	0.000009
	cci-miR159e	TTTGGATTGAAAGGAGCTCT	20	sof-miR159e	0	57	0.0000006
	cci-miR319a-5p	AGAGCTTCCTTCAGTCCACT	20	mtr-miR319a-5p	0	31	0.000001
	cci-miR319b-3p	TTGGACTGAAGGGTGCTCCCT	21	bdi-miR319b-3p	0	24	0.0000001
	cci-miR319c-5p	GAGCTTCCTTCAGCCCACTC	20	sly-miR319c-5p	0	9	0.0000006
	cci-miR159c	TTGGATTGAAGGGAGCTCCA	20	osa-miR159c	0	8	0.0000006
	cci-miR159	TTTGGTTTGAAGGGAGCTCTA	21	pde-miR159	0	5	0.0000002
	cci-miR159b	TTGGATTGAAGGGAGCTCTG	20	osa-miR159b	0	4	0.0000006
	cci-miR159c	TTTGGATTGAAGGGAGCTCC	20	ath-miR159c	0	3	0.0000006
	cci-miR159a-5p	GAGCTCCCTGAAGTCCAAT	19	gma-miR159a-5p	1	2	0.0006
	cci-miR159d	AGCTGCTGAGCTATGGATC	19	gma-miR159d	1	1	0.0005
	cci-miR319c	TGGACTGAAAGGAGCTCC	18	gma-miR319c	0	1	0.000008
	cci-miR319g	TTGGACTGAAGGGAGCTCCT	20	gma-miR319g	0	1	0.0000006
	cci-miR159c	AAAGGAGAGGAGAAGGAA	18	mdm-miR159c	0	1	0.00001
MIR160	cci-miR160h	TGCCTGGCTCCCTGCATGCCA	21	ptc-miR160h	0	149	0.0000003
	cci-miR160a-3p	GCGTATGAGGAGCCAAGCATA	21	gma-miR160a-3p	0	7	0.0000002
	cci-miR160d	TGCCTGGCTCCCTGAATGCCA	21	cpa-miR160d	0	1	0.0000002
	cci-miR160g	TGCCTGGCTCCCTGTATGCCAT	22	mes-miR160g	0	1	0.00000007
MIR164	cci-miR164b-5p	TGGAGAAGCAGGGCACGTGCA	21	ath-miR164b-5p	0	1596	0.0000003
	cci-miR164d	TGGAGAAGCAGGGCACATGCT	21	mtr-miR164d	0	116	0.0000002
	cci-miR164g-3p	CACGTGCTCCCCTTCTCCA	19	zma-miR164g-3p	0	36	0.000002
	cci-miR164b	TGGAGAAGCAGGGCACGT	18	gma-miR164b	0	10	0.000009
	cci-miR164d	TGGAGAAGCAGGGCACGTGCT	21	osa-miR164d	0	3	0.0000002
	cci-miR164e-5p	TGGAGAAGCAGGGCACGTGCAA	22	bra-miR164e-5p	0	1	0.00000007
	cci-miR164c	TGGAGAAGCAGGGAACGTGCA	21	osa-miR164c	1	1	0.00004
MIR164_2	cci-miR164b	TGGAGAAGCAGGGCACTT	18	far-miR164b	0	3	0.000009
MIR166	cci-miR166c	TCGGACCAGGCTTCATTCCTC	21	mtr-miR166c	0	140263	0.0000004
	cci-miR166u	TCTCGGACCAGGCTTCATT	19	gma-miR166u	0	2404	0.000005
	cci-miR166c-5p	GGAATGTTGTCTGGCTCGAGG	21	gma-miR166c-5p	0	1176	0.0000004
	cci-miR166a	TCGGACCAGGCTTCATTCCCC	21	pta-miR166a	0	234	0.0000003
	cci-miR166p	TCGGACCAGGCTCCATTCCT	20	ptc-miR166p	0	98	0.0000006
	cci-miR166d-5p	GAATGTCGGCTGGTTCGAGA	20	stu-miR166d-5p	1	85	0.0001
	cci-miR166e-3p	TCGAACCAGGCTTCATTCCCC	21	osa-miR166e-3p	0	51	0.0000002
	cci-miR166d	TCGGGCCAGGCTTCATTCCCC	21	mtr-miR166d	1	48	0.00004
	cci-miR166i-5p	AATGAAGTTTGATCCAAGATC	21	osa-miR166i-5p	1	47	0.00007

	cci-miR166q	TCGGACCAGGCTTCATTCCTT	21	ptc-miR166q	0	21	0.0000002
	cci-miR166i	TCGGACCAGGCTTCATTCT	19	cme-miR166i	0	20	0.000002
	cci-miR166m	TCGGACCAGGCATCATTCC	19	ppt-miR166m	0	20	0.000002
	cci-miR166k-3p	TCGGACCAGGCTTCAATCCCT	21	osa-miR166k-3p	0	12	0.0000002
	cci-miR166i-5p	GGAATGCCGTCTGGTTCGAG	20	gma-miR166i-5p	1	11	0.0002
	cci-miR166b-5p	GGAATGTTGTCTGGCTCG	18	osa-miR166b-5p	0	9	0.000009
	cci-miR166m	GGACCATGCTTCATTCCCC	19	gma-miR166m	1	7	0.0006
	cci-miR166a	TCGGACCAGGCTTCATTCCCCC	22	csi-miR166a	0	6	0.00000008
	cci-miR165b	TCGGACCAGGCTTCATGCCCC	21	ath-miR165b	1	5	0.00004
	cci-miR166a-5p	GGAATGTTGTCTGGTTCAAGG	21	osa-miR166a-5p	0	5	0.0000002
	cci-miR166i-3p	TCGGAGCAGGCTTCATTCCTC	21	osa-miR166i-3p	1	3	0.00004
	cci-miR166b	TCGGGCCAGGCTTCATTCCT	20	mtr-miR166b	1	2	0.0001
	cci-miR166e-5p	GGAATGTTGGCTGGTTCGAG	20	mtr-miR166e-5p	1	2	0.0001
	cci-miR166c-5p	TCGGACCAGGCAACATTCC	19	osa-miR166c-5p	1	2	0.0006
	cci-miR166a-5p	GGACTGTCGTCTGGCTCGAGG	21	ath-miR166a-5p	1	1	0.00004
	cci-miR166e-5p	GGAATGTTGTCTGGCACGAGG	21	ath-miR166e-5p	0	1	0.0000002
	cci-miR166e-3p	CTCGGACCAGGCTTCATTCCC	21	bdi-miR166e-3p	0	1	0.0000002
	cci-miR166b	TCGGGCCAGGCTTCATTCCC	20	csi-miR166b	1	1	0.0001
	cci-miR166h-5p	GGAATGTTGTGTGGCTCGAGG	21	gma-miR166h-5p	1	1	0.00004
MIR167	cci-miR167h	TTGAAGCTGCCAGCATGA	18	gma-miR167h	0	9	0.000009
MIR167_1	cci-miR167d	TGAAGCTGCCAGCATGATCTGG	22	ath-miR167d	0	21213	0.00000009
	cci-miR167c-5p	TGAAGCTGCCAGCATGATCTGC	22	tae-miR167c-5p	0	2262	0.00000008
	cci-miR167d	TGAAGCTGCCAGCATGATCTGA	22	cpa-miR167d	0	1244	0.00000007
	cci-miR167c-3p	GGTCATGCTCGGACAGCCTCACT	23	stu-miR167c-3p	0	81	0.00000001
	cci-miR167b	TGAAGCTGCCAGCATGATCTAA	22	bna-miR167b	0	76	0.00000006
	cci-miR167a	TCAGATCATCTTGCAGCTTCA	21	gra-miR167a	0	58	0.0000003
	cci-miR167i	TGAAGCTGCCAGCATGATCTTA	22	mdm-miR167i	0	18	0.00000004
	cci-miR167d-3p	GATCATGTGGTAGCTTCACC	20	stu-miR167d-3p	1	18	0.0002
	cci-miR167h-5p	TGAAGCTGCCAACATGATCTG	21	ptc-miR167h-5p	0	9	0.0000001
	cci-miR167d	TGAAGCAGCCAGCATGATC	19	bna-miR167d	1	3	0.0006
	cci-miR167h-3p	AGGTCATGCTGTAGTTTCATC	21	osa-miR167h-3p	0	3	0.0000002
	cci-miR167b	TGAAGCTGACAGCATGATCTA	21	tae-miR167b	0	3	0.0000002
	cci-miR167f-3p	TGAAGCTGCAGCATGATCTG	20	ata-miR167f-3p	0	1	0.0000006
MIR168	cci-miR168c-5p	TCGCTTGGTGCAGGTCGGGAC	21	bra-miR168c-5p	0	694	0.0000004
	cci-miR168	TCCCGCCTTGCATCAATTGAAT	22	aau-miR168	1	13	0.000009
	cci-miR168a-5p	TCGCTTGGTGCAGATCGGGAC	21	osa-miR168a-5p	0	13	0.0000002
	cci-miR168b-3p	CCCGTCTTGTATCAATTGAAT	21	ath-miR168b-3p	1	5	0.00004
	cci-miR168a-3p	CCCGGCTTGCACCAAGTGAAT	21	osa-miR168a-3p	1	3	0.00004
	cci-miR168b-5p	TCGCTTGGTGCAGGTCGGGA	20	ath-miR168b-5p	0	2	0.0000007
	cci-miR168b-3p	CCCGTCTTGCATCAAGTGAA	20	zma-miR168b-3p	1	1	0.0001
MIR169_1	cci-miR169k	TAGCCAAGGATGACTTGCCTGC	22	bna-miR169k	0	232	0.00000008
	cci-miR169n	TAGCCAAGAATGACTTGCCT	20	osa-miR169n	0	52	0.0000006
	cci-miR169h	TAGCCAAGGATGACTTGCCTG	21	ath-miR169h	0	3	0.0000002
	cci-miR169f	TGAAGAGAAGAGTGTTGTTT	20	mdm-miR169f	1	2	0.0001

	cci-miR169ab	AGCCAAGGAAGACTTGCC	18	ptc-miR169ab	0	1	0.000009
	cci-miR169q	TAGCCAAGGACGACTTGCCTG	21	ptc-miR169q	0	1	0.0000002
	cci-miR169e-5p	TAGCCAATGATGACTTGCCT	20	sly-miR169e-5p	1	1	0.0001
	cci-miR169f	CCAAGAATGACTTGCCTG	18	tcc-miR169f	0	1	0.000009
MIR169_2	cci-miR169a-5p	CAGCCAAGGATGACTTGCCGA	21	ath-miR169a-5p	0	91	0.0000001
	cci-miR169r-3p	GGCAAGTTGTCTTTGGCTACA	21	zma-miR169r-3p	1	45	0.00004
	cci-miR169a-3p	GGCAAGTTGTCTTTGGCTAC	20	ath-miR169a-3p	1	11	0.0001
	cci-miR169b-5p	CAGCCAAGGATGACTTGCCG	20	ath-miR169b-5p	0	1	0.0000006
MIR171_1	cci-miR171b-3p	TTGAGCCGTGCCAATATCACG	21	ath-miR171b-3p	0	879	0.0000003
	cci-miR171d-5p	TTGGCCGGGCTCACTCAGA	19	osa-miR171d-5p	1	470	0.0007
	cci-miR171d	TGATTGAGCCGTGCCAATATC	21	cpa-miR171d	0	172	0.0000003
	cci-miR171k-3p	TTGAGCCGCGCCAATATCACT	21	gma-miR171k-3p	0	163	0.0000001
	cci-miR171e	GATATTGGTCCGGTTCAATAA	21	ppe-miR171e	0	69	0.0000002
	cci-miR171a	TTGAGCCGCGTCAATATCTCC	21	mdm-miR171a	0	50	0.0000003
	cci-miR171b-5p	TGTTGGAATGGCTCAATCAA	20	ata-miR171b-5p	1	32	0.0001
	cci-miR171a	TTGAGTCGTGCCAATATC	18	mtr-miR171a	0	2	0.000009
	cci-miR171a-3p	TGATTGATCCGCGCCAATATC	21	ath-miR171a-3p	1	1	0.00004
	cci-miR171g	TGAGCCGCGCCAATATCT	18	bna-miR171g	0	1	0.000009
	cci-miR171l	CGATGTTGGTGAGGTTCAATC	21	gma-miR171l	0	1	0.0000002
	cci-miR171e	TTGAGCCGCGTCAATATC	18	sly-miR171e	0	1	0.000009
	cci-miR171b-5p	TTGAACCGCGCCAATATC	18	zma-miR171b-5p	0	1	0.000009
MIR172	cci-miR172c-5p	GTAGCATCATCAAGATTCACA	21	mtr-miR172c-5p	0	217	0.0000004
	cci-miR172d-5p	AGCACCATCAAGATTCACA	19	zma-miR172d-5p	0	174	0.000002
	cci-miR172a	AGAATCTTGATGATGCTGCAT	21	ath-miR172a	0	38	0.0000002
	cci-miR172a	CTGCAGCATCATCAGGATT	19	mtr-miR172a	0	1	0.000003
MIR319	cci-miR319i	TTGGGCTGAAGGGAGCTCCC	20	ptc-miR319i	0	7	0.0000006
MIR390	cci-miR390b	AAGCTCAGGAGGGATAGCGCC	21	ppt-miR390b	0	196	0.0000003
	cci-miR390a-3p	CGCTATCTATCCTGAGTTTCA	21	ath-miR390a-3p	1	63	0.00005
	cci-miR390	AAGCACAGGATGGATAGCG	19	pta-miR390	1	14	0.0006
	cci-miR390.1	CGCTATCCCTCCTGAGCTTTA	21	atr-miR390.1	0	1	0.0000002
	cci-miR390-3p	GCTATCTATCCTGAGCTCC	19	osa-miR390-3p	0	1	0.000002
MIR393	cci-miR393a-5p	TCCAAAGGGATCGCATTGATCC	22	ath-miR393a-5p	0	66	0.00000007
	cci-miR393	TCCAAAGGGATCGCATTGAT	20	ghr-miR393	0	23	0.0000006
	cci-miR393c-3p	ATCATGCTATCCCTTTGGATT	21	gma-miR393c-3p	0	19	0.0000003
	cci-miR393	ATCCAAAGGGATCGCATTGATC	22	csi-miR393	0	4	0.00000004
	cci-miR393a-3p	TCATGCTATCTCTTTGGATT	20	ath-miR393a-3p	0	1	0.0000006
MIR3932	cci-miR3932a	TCGTCGTCATCACAAAGTT	19	ath-miR3932a	1	1	0.0006
MIR394	cci-miR394b-5p	TTGGCATTCTGTCCACCTCC	20	ath-miR394b-5p	0	119	0.0000006
MIR395	cci-miR395b	CTGAAGTGTTTGGGGGAACTCC	22	sly-miR395b	0	821	0.00000004
	cci-miR395o	GAGTTCCTCCAAACACTT	18	osa-miR395o	0	3	0.000009
	cci-miR395a	CTGAAGTGTTCGGGGGAACTC	21	ath-miR395a	1	2	0.00004
	cci-miR395i	CTGAAGTGTTTGGAGGAACTC	21	aly-miR395i	0	1	0.0000002
	cci-miR395i	AAGTGTTTGGGGGAACTC	18	gma-miR395i	0	1	0.000009
MIR396	cci-miR396f	TTCCACGGCTTTCTTGAACTG	21	ptc-miR396f	0	41200	0.0000004

	cci-miR396	TTCCACAGCTTTCTTGAACTT	21	pta-miR396	0	28712	0.0000004
	cci-miR396a-3p	GTTCAATAAAGCTGTGGGAAG	21	ath-miR396a-3p	0	6661	0.0000003
	cci-miR396a-3p	GTTCAAGAAAGCTGTGGGAA	20	cca-miR396a-3p	0	2188	0.0000006
	cci-miR396a-3p	TTCAATAAAGCCGTGGGAAG	20	gma-miR396a-3p	1	699	0.0001
	cci-miR396c	TTCAAGAAAACTGTGGGAAG	20	csi-miR396c	1	646	0.0002
	cci-miR396a-5p	TTCCACAGCTTTCTTGAACTG	21	ath-miR396a-5p	0	156	0.0000003
	cci-miR396a	CAGCTTTCTTGAACTTTCT	19	hbr-miR396a	0	90	0.000002
	cci-miR396-3p	CCACAGCTTTCTTGAGCTT	19	ama-miR396-3p	0	27	0.000002
	cci-miR396g-5p	TTCCACGGCTTTCTTGAACTT	21	ptc-miR396g-5p	0	23	0.0000002
	cci-miR396e	TCCACAGGCTTTCTTGAACTG	21	sbi-miR396e	0	10	0.0000002
	cci-miR396c	TTCAAGAAAACTGTGGGAA	19	cca-miR396c	1	7	0.0007
	cci-miR396b-3p	TTCAATAAAGCCGTGGGAA	19	osa-miR396b-3p	1	7	0.0006
	cci-miR396g-5p	CAGTTCAATAAAGCCGTGGGA	21	zma-miR396g-5p	1	6	0.00004
	cci-miR396j	TTCCACAGCTATCTTGAA	18	gma-miR396j	0	4	0.000009
	cci-miR396b	TTCCACAGCTTTCTTGAACT	20	vvi-miR396b	0	4	0.000001
	cci-miR396	GTTCAAAAAAGCCGTGGGA	19	pde-miR396	1	3	0.0006
	cci-miR396	GTTCAATAAAGCCGTGGAA	19	smo-miR396	1	3	0.0006
	cci-miR396e-3p	TCAAGAAAGCCGTGGGAAG	19	zma-miR396e-3p	0	3	0.000002
	cci-miR396f	AGCTTTCTTGAACTTCTT	18	gma-miR396f	0	2	0.000009
	cci-miR396-3p	TCCAAGAAAACTGTGGGAA	19	stu-miR396-3p	1	2	0.0005
	cci-miR396g-3p	TCTTCCACAGCTTTCTTGAAC	21	zma-miR396g-3p	0	2	0.0000002
	cci-miR396a	TCCACAGCTTTCTTGAACAG	20	mdm-miR396a	0	1	0.0000006
	cci-miR396c-3p	GTCAAGAAAGCTGTGGGAAG	20	osa-miR396c-3p	0	1	0.0000006
MIR397	cci-miR397	ATTGAGTGCAGCGTTGATGA	20	lja-miR397	0	21	0.0000006
MIR398	cci-miR398a-3p	TGTGTTCTCAGGTCACCCCTT	21	ath-miR398a-3p	0	85	0.0000002
	cci-miR398c	TGTGTTCTCAGGTCGCCCCTG	21	gma-miR398c	0	81	0.0000001
	cci-miR398a-5p	GGAGTGTCATGGGAACACA	19	aly-miR398a-5p	1	4	0.0006
	cci-miR398b-3p	TTGTGTTCTCAGGTCACCCCT	21	stu-miR398b-3p	0	1	0.0000002
MIR399	cci-miR399i	TGCCAAAGGAGAATTGCCCTG	21	gma-miR399i	0	15	0.0000002
	cci-miR399i	TGCCAAAGGAGAGTTGCCCTA	21	ptc-miR399i	0	15	0.0000002
	cci-miR399d	TGCCAAAGGAGATTTGCCCCG	21	ath-miR399d	0	1	0.0000002
	cci-miR399o	TGCCAAAGGAGAGCTGCCCTG	21	mtr-miR399o	0	1	0.0000002
MIR403	cci-miR403-3p	TTAGATTCACGCACAAACTCG	21	ath-miR403-3p	0	4500	0.0000003
	cci-miR403a	TTAGATTCACGCACAAACTT	20	gma-miR403a	0	471	0.0000009
	cci-miR403-5p	TTAGATTCACGCACAAAA	18	bra-miR403-5p	0	7	0.00001
	cci-miR403-5p	GTTTGTTCGTGAATCTAACA	20	sly-miR403-5p	1	6	0.0001
	cci-miR403-3p	TAGATTCACGCACAAGCTCG	20	sly-miR403-3p	0	2	0.0000006
MIR408	cci-miR408	TGCACTGCCTCTTCCCTGG	19	cpa-miR408	0	35	0.000003
MIR414	cci-miR414	TGACGATGATGATGATGATG	20	ath-miR414	1	1	0.0002
MIR437	cci-miR437x-5p	GTTTGACTTAGGACAACTCTA	21	sbi-miR437x-5p	0	2	0.0000002
MIR444	cci-miR444b.2	TGCAGTTGTTGTCTCAAGCTT	21	osa-miR444b.2	0	2	0.0000002
	cci-miR444b	CTTGAGACAGCAACTGCA	18	hvu-miR444b	0	1	0.00001
	cci-miR444d.3	TTGTGGCTTTCTTGCAAGTTG	21	osa-miR444d.3	0	1	0.0000002
	cci-miR444f	TGCAGTTGTTGCCTCAAGCTT	21	osa-miR444f	0	1	0.0000002

MIR477	cci-miR477-5p	ACTCTCCCTCAAAGGCTTC	19	ppe-miR477-5p	0	455	0.000005
	cci-miR477a	ACTCTCCCTCAAGGGCTTCTG	21	nta-miR477a	0	265	0.0000003
	cci-miR477a-5p	TCTCCCTCAGAGGCTTCC	18	ptc-miR477a-5p	0	1	0.000009
MIR482	cci-miR2118	TTTCCTATTCCACCCATCCCAT	22	pgi-miR2118	0	43	0.00000004
MIR529	cci-miR529-5p	AGAAGAGAGAGAGTACAGCCT	21	zma-miR529-5p	0	1	0.0000002
MIR774	cci-miR774b-5p	GTCATCCAAACCTTCATCT	19	aly-miR774b-5p	1	1	0.0006
MIR818	cci-miR1130b-3p	TTATATTAAGGGACGGAGG	19	tae-miR1130b-3p	1	15	0.0007
	cci-miR1436	ACATTATGAGACGGAGGGAGT	21	osa-miR1436	1	2	0.00004
	cci-miR1439	TTTTGGGACGGAGTGAGTA	19	osa-miR1439	1	1	0.0006
	cci-miR1130a	TTACATTAAGAGACGGAGG	19	tae-miR1130a	1	1	0.0006
MIR821	cci-miR821d	CAACTTTGTTGTTGTTGAC	19	sbi-miR821d	1	1	0.0009
	cci-miR821e	AAGTCATCAAAACAAAAGT	19	sbi-miR821e	1	1	0.0006
MIR827	cci-miR827	TTAGATGATCATCAGCAAACA	21	osa-miR827	1	5537	0.00007
	cci-miR827-5p	TTTGTTGGTGGTCATCTAA	19	bdi-miR827-5p	1	251	0.0007
	cci-miR827	TTAGATGAACATCAGCAAACA	21	nta-miR827	1	3	0.00004
MIR828	cci-miR828	TCTTGCTTAAATGAGTGTTCCA	22	ath-miR828	1	24	0.000009
MIR834	cci-miR834	TGGTAGCAGTAGTGGTGGT	19	ath-miR834	1	2	0.0006
MIR835	cci-miR835-5p	TTCTTGCATATGTTCTTT	18	ath-miR835-5p	0	1	0.000009
MIR845_3	cci-miR845b	CAATTGGTATCAGAGCTA	18	vvi-miR845b	0	2	0.00001
MIR858	cci-miR858a	TTTCGTTGTCTGTTCGACCTT	21	ath-miR858a	0	1090	0.0000002
	cci-miR858b	TTCGCTGTCTGTTCGACCTTG	21	ath-miR858b	1	3	0.00004
	cci-miR858-3p	TTCGTTGTCTGCTCGACC	18	aly-miR858-3p	0	2	0.000009
	cci-miR858	TCGTTGTCTGGTCGACCTTG	20	ppe-miR858	1	1	0.0001
MIR862	cci-miR862-3p	ATATGCTGGATTTACTTGAAG	21	ath-miR862-3p	1	1	0.00004
MIR1120	cci-miR1120a	TTATATTATGAGACGGAG	18	tae-miR1120a	0	5	0.00001
MIR1122	cci-miR1133	GGACGGAGGGAGTATATG	18	tae-miR1133	0	3	0.00002
	cci-miR5281e	ATAAATAGAACCGGAGGGAG	20	mtr-miR5281e	1	2	0.0001
MIR1511	cci-miR1511	ACCTAGCTCTGATACCATGA	20	mdm-miR1511	0	2	0.0000006
MIR1520	cci-miR1520q	ACCAATTAGAACATGACACA	20	gma-miR1520q	1	1	0.0002
MIR1863	cci-miR1863b	AGCTCTGATACCATATTAACTG	22	osa-miR1863b	1	4	0.00001
MIR2084	cci-miR2084	CCTGCATTGGTGGATTGTG	19	ppt-miR2084	1	1	0.0006
MIR2275	cci-miR2275b-3p	AGATATTAGAGAAAACTGA	19	zma-miR2275b-3p	1	3	0.0007
MIR2275	cci-miR2275d-5p	AGAGTTGGAGTAAAGAAAA	19	zma-miR2275d-5p	1	1	0.0006
MIR2646	cci-miR2646b	ATGACATGTAGTGATGATGT	20	mtr-miR2646b	1	1	0.0002
MIR2673	cci-miR2673b	CCTCTTCCTCTTCCTCTTCC	20	mtr-miR2673b	0	2	0.0000009
MIR2912	cci-miR2912a	TCTAGAACTCCAGATATGG	19	peu-miR2912a	1	3	0.0006
MIR3630	cci-miR3630-3p	TGGGAATCTCTTTGATGCAC	20	vvi-miR3630-3p	1	3	0.0003
MIR5067	cci-miR5181-3p	CACTTATTTTGGAACGGAGGG	21	ata-miR5181-3p	1	4	0.00004
	cci-miR5049d	ACAACTATTTAGGAACGGAG	20	hvu-miR5049d	1	3	0.0002
	cci-miR5181-5p	GACAATTATTCTGGATCGG	19	ata-miR5181-5p	1	1	0.0006
	cci-miR5174d-3p	TTATGGAACGGAGAGAGT	18	bdi-miR5174d-3p	0	1	0.00001
	cci-miR5049b	TATTTAGGGACAGAGGGAG	19	hvu-miR5049b	1	1	0.0006
	cci-miR5049c	GACAATTATTTTGGGACAGAGG	22	hvu-miR5049c	1	1	0.00001
MIR5298	cci-miR5298a	TTCTTCATCTTCATCTCAT	19	mtr-miR5298a	1	1	0.0006

MIR5387	cci-miR5387b	CTTTAGCACCGGCCAGAGCCAC	22	sbi-miR5387b	1	1	0.00001
MIR5564	cci-miR5564a	TGGGGAAGCAATTCGTCGAACA	22	sbi-miR5564a	0	23	0.00000004
	cci-miR5564b	GCAATTCGTCGAACAGCTTG	20	sbi-miR5564b	0	15	0.0000006
MIR5565	cci-miR5565b	TCGCATCAATCCACATGTGTT	21	sbi-miR5565b	1	1	0.00004
MIR5568	cci-miR5568d-5p	TGGCTTTTCTAGACACATAGC	21	sbi-miR5568d-5p	1	1	0.00004
MIR6161	cci-miR6161b	TGGACCAGTATACTTTGCT	19	nta-miR6161b	1	1	0.0006
MIR6476	cci-miR6476a	TCAGTGGAGATGAAACATG	19	ptc-miR6476a	0	97	0.000005
MIR7982	cci-miR7982a	TGGAGGATAATAATATATA	19	stu-miR7982a	1	1	0.0006
MIR7996	cci-miR7996b	TGGTATATATGAAATTTGAA	20	stu-miR7996b	1	1	0.0002
MIR8762	cci-miR8762c	CAACAAAGTTAGCAAACGT	19	gra-miR8762c	1	2	0.0005
NA	cci-miR6478	CCGACCTTAGCTCAGTTGGT	20	ptc-miR6478	0	5673	0.000001
	cci-miR6300	GTCGTTGTAGTATAGTGG	18	gma-miR6300	0	4672	0.00002
	cci-miR894	CGTTTCACGTCGGGTTCACC	20	ppt-miR894	0	744	0.000001
	cci-miR8175	GATCCCCGGCAACGGCGCCA	20	ath-miR8175	0	233	0.000001
	cci-miR5072	TTCCCCAGCGGAGTCGCCA	19	osa-miR5072	0	41	0.000005
	cci-miR1873	TCAACATGGTATCAGAGCT	19	osa-miR1873	0	29	0.000003
	cci-miR845	TGCTCTGATACCAATTGTTG	20	bdi-miR845	0	22	0.000001
	cci-miR5141	TTATCTGTCAGTCGCGTCGGGTCT	24	rgl-miR5141	0	22	0.000000006
	cci-miR5523	CTAGTAAATACGTTCCTCCTCA	22	osa-miR5523	1	10	0.00002
	cci-miR408	TGCACTGCCTCTTCCCTGGCT	21	smo-miR408	0	8	0.0000002
	cci-miR9493	ATTATGAAACGGAGGGAGTA	20	bdi-miR9493	1	7	0.0003
	cci-miR5561-5p	ATTTGGAGAGACATTGACA	19	mtr-miR5561-5p	1	7	0.0006
	cci-miR5174e-3p.2	TTATGAAACGGAGGGAGTAG	20	bdi-miR5174e-3p.2	1	5	0.0002
	cci-miR5538	ACTGAACTCAATCACTTGCTGC	22	osa-miR5538	0	5	0.00000008
	cci-miR6456	GGATCTAATGCAAGGACTC	19	ptc-miR6456	1	5	0.001
	cci-miR5139	AAACCTGGCTCTGATACCA	19	rgl-miR5139	0	5	0.000004
	cci-miR8590	GATTTGTAGAAAAAAAAA	18	atr-miR8590	0	4	0.00002
	cci-miR156r	CTGACAGAAGATAGAGAGCA	20	gma-miR156r	0	4	0.0000006
	cci-miR5368	GGACAGTCTCAGGTAGACA	19	gma-miR5368	0	4	0.000003
	cci-miR5293	CTTCATTCCACTTCTTCATC	20	mtr-miR5293	1	4	0.0002
	cci-miR5655	GTAGACACATAAGTAGGAG	19	ath-miR5655	1	3	0.0006
	cci-miR5721	CATTTCTCATTCCATTTTT	19	bra-miR5721	1	3	0.001
	cci-miR8744	AAAAAATGGACAAAGTAGT	19	gra-miR8744	1	3	0.0006
	cci-miR1056	TGGATCTTTGAATCATAAC	19	ppt-miR1056	1	3	0.0007
	cci-miR3627b	TCGCAGGAAAGATGGCGCT	19	ptc-miR3627b	1	3	0.0005
	cci-miR1118	TCCCTCCATTCCTTAATGT	19	tae-miR1118	1	3	0.0006
	cci-miR5658	TGATGATGATAATGATGAA	19	ath-miR5658	1	2	0.0009
	cci-miR7782-3p	TGCTCTGATACCATGTTG	18	bdi-miR7782-3p	0	2	0.00001
	cci-miR4394	TGGACTAAAGAGAAAGGGG	19	gma-miR4394	0	2	0.000003
	cci-miR5041-3p	TTCATCTTCAACTTTCTCA	19	gma-miR5041-3p	1	2	0.0008
	cci-miR8705	ACCCACGAACTTGTATGAA	19	gra-miR8705	1	2	0.0006
	cci-miR6485	TAGGATGTAGAAGATCATAA	20	hbr-miR6485	1	2	0.0003
	cci-miR3711	AGGCCCTCCTTCTAGCGCCA	20	pab-miR3711	0	2	0.0000006
	cci-miR172i	TGCAGCATCATCAGGATT	18	ptc-miR172i	0	2	0.00001

cci-miR6233-3p	TTTGGTTTTGGTAATTAATG	20	sbi-miR6233-3p	0	2	0.0000006
cci-miR1128	TTTGGGACGGAGGGAGTAGTA	21	ssp-miR1128	0	2	0.0000002
cci-miR8035	GAAAGTGATAGTGAAGATG	19	stu-miR8035	1	2	0.0006
cci-miR4233	GAGTTGATGTGGATGATGT	19	aly-miR4233	1	1	0.0006
cci-miR1886.1	TTTCATCTCTCTTCTCTCA	19	ath-miR1886.1	1	1	0.0006
cci-miR5653	CAACTCAACTCAACACAAC	19	ath-miR5653	1	1	0.0006
cci-miR8605	TTAATCTGGACCATTGGAT	19	atr-miR8605	1	1	0.0006
cci-miR5174e-5p.2	TTATGGGACAGAGGGAGTA	19	bdi-miR5174e-5p.2	1	1	0.0006
cci-miR5176-3p	GTGATGAGGTGGCATAGAAT	20	bdi-miR5176-3p	1	1	0.0002
cci-miR5281b	CCTCCGTTTCTATTTATAA	19	bdi-miR5281b	1	1	0.0006
cci-miR7711-5p.4	TATCTTGGTCATAATATTCA	20	bdi-miR7711-5p.4	1	1	0.0002
cci-miR7731-5p	GTTTGCTCAGAAATTTGGA	19	bdi-miR7731-5p	1	1	0.0006
cci-miR6034	TGATGTTTATAGCTTTGGG	19	bna-miR6034	1	1	0.001
cci-miR1507c-3p	GATGATGCTTGGAATGAGG	19	gma-miR1507c-3p	1	1	0.0006
cci-miR1511	CATGGTATCAGAGCTTGGTT	20	gma-miR1511	1	1	0.0002
cci-miR167k	TGAAGCTGCCAGCCTGATCT	20	gma-miR167k	0	1	0.0000006
cci-miR4995	AGGCAGTGGCTTGGTTAAGGG	21	gma-miR4995	0	1	0.0000003
cci-miR5380c	CTCTTCATCTTCACAATTCAT	21	gma-miR5380c	1	1	0.00004
cci-miR9742	TGTTGTTTGTTTTGAAGCA	19	gma-miR9742	1	1	0.0006
cci-miR8689	TGCTCATGGGTTGGGTCGG	19	gra-miR8689	1	1	0.0006
cci-miR8702	GGTATTTTCTCTGGGGAAGG	20	gra-miR8702	1	1	0.0001
cci-miR8756	TTAAAATTTTAACCGTCCA	19	gra-miR8756	1	1	0.0006
cci-miR6173	GCCGTAAACGATGGATAC	18	hbr-miR6173	0	1	0.00002
cci-miR6482	GGAACTGGTATCAACCCAGC	20	hbr-miR6482	0	1	0.000001
cci-miR6196	CTCTCCATCTGCTCGTCCT	19	hvu-miR6196	1	1	0.0008
cci-miR5205a	TACAATTTGGGATGGAGGG	19	mtr-miR5205a	1	1	0.0006
cci-miR5205c	TAATTAGGGACGGAGGTAGT	20	mtr-miR5205c	0	1	0.0000006
cci-miR1854-5p	GGTGAAATTTGTGGATTGG	19	osa-miR1854-5p	1	1	0.0006
cci-miR2120	CAACCAGGACTAAAGATCT	19	osa-miR2120	1	1	0.0006
cci-miR3705	TAAGTGGTTATAATCTGGA	19	pab-miR3705	1	1	0.0006
cci-miR8127-5p	AACTGTGTACATACCCTTT	19	ppe-miR8127-5p	1	1	0.0006
cci-miR6443	CTCCATCATCTATTATCATA	20	ptc-miR6443	1	1	0.0001
cci-miR6470	TTTTAAGATGATATCAGAG	19	ptc-miR6470	1	1	0.0006
cci-miR5382	CCAATCTAAACAGGCCCT	18	sbi-miR5382	0	1	0.000009
cci-miR6220-5p	CTTATAATTTAGAATGGAG	19	sbi-miR6220-5p	1	1	0.0006
cci-miR6235-5p	TGAGAGAAAAACACTGTTG	19	sbi-miR6235-5p	1	1	0.0006
cci-miR1112-3p	GAATGCTATACCAAAGTCA	19	smo-miR1112-3p	1	1	0.0006
cci-miR8044-5p	TCAAATATTGTTGGAGATG	19	stu-miR8044-5p	1	1	0.0006
cci-miR1134	AACAAAAAGAAGAAGAAGA	19	tae-miR1134	1	1	0.0009
cci-miR9773	TTTGTTTTTATGTTATTTTGTGA	23	tae-miR9773	0	1	0.00000001
cci-miR9774	CAAGATATTGGGTATTTC	18	tae-miR9774	0	1	0.00001
cci-miR156h	ATGCTTTCTCTCTTCTGTC	19	vvi-miR156h	1	1	0.0006
cci-miR3625-3p	CTTCCAGTATTCATCTCCC	19	vvi-miR3625-3p	1	1	0.0006

Table S2: Sequence of primers of C. cyminum novel miRNAs.

Novel miRNA	Specific primer sequence
cci-miRN3-5p	TAGAAGTGTGACCTGTCTTGCA
cci-miRN19-3p	CTGAGCCGAACCAATATTACTC
cci-miRN22-3p	TGGTGCCACACTGCTCGCGTTT
cci-miRN32-5p	GTTCCCTTGACCACTTCATTGG
cci-miRN34-3p	TACATTGAGGGAAATTGAGGGA
